# Photoinitiated Degradation Kinetics of the Organic UV Filter Oxybenzone in Solutions and Aerosols: Impacts of Salt, Photosensitizers, and the Medium

**DOI:** 10.1021/acsestair.4c00149

**Published:** 2024-10-16

**Authors:** Adam Cooper, Alexis Shenkiryk, Henry Chin, Maya Morris, Lincoln Mehndiratta, Kanuri Roundtree, Tessa Tafuri, Jonathan H. Slade

**Affiliations:** † Department of Chemistry & Biochemistry, University of California, San Diego, La Jolla, California 92093, United States; ‡ Department of Chemistry & Biochemistry, University of California, Los Angeles, Los Angeles, California 90095, United States

**Keywords:** photochemistry, aerosol, kinetics, photosensitizer, sea spray aerosol, sunscreen

## Abstract

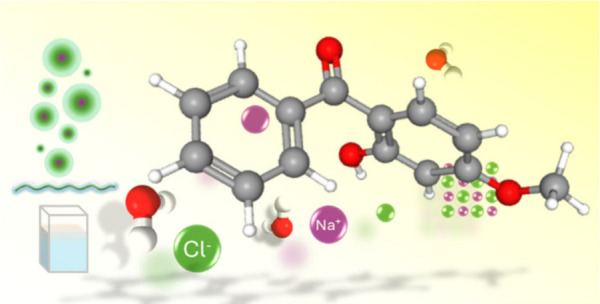

Organic UV filters like oxybenzone (BP3) in sunscreens are seawater pollutants suspected to transfer to the atmosphere via sea spray aerosol (SSA). This study examines the photoinitiated degradation of BP3 in artificial and real seawater compared to SSA mimics containing NaCl and 4-benzoylbenzoic acid (4-BBA). We investigated pure, binary, and ternary mixtures of BP3, NaCl, and 4-BBA using solar-simulated light to isolate the effects of salt and photosensitization on BP3 degradation. Results showed significantly faster degradation in the aerosol phase (*J*_eff,env_ ≈ 10^–3^–10^–2^ s^–1^ or *t*_1/2_ < 10 min) compared to bulk solutions (*J*_eff,env_ ≈ 10^–6^ s^–1^ or *t*_1/2_ > 1 day). The photosensitizer enhanced BP3 photodegradation in both phases more than when mixed with salt or all three components in solutions. BP3 photodegradation was most enhanced by salt in the aerosol phase. High-resolution molecular analysis via Orbitrap LC-MS/MS revealed more acutely toxic compounds (benzophenone, benzoic acid, and benzaldehyde) in irradiated aerosols than in solution, supported by electronic structure and toxicity modeling. These findings highlight that seawater may serve as a reservoir for BP3 and other organic UV filters and that upon transfer into SSA, BP3 rapidly transforms, increasing aerosol toxicity.

## Abstract 2

When exposed to solar-simulated light, oxybenzone (a sunscreen active ingredient) is long-lasting in seawater but rapidly degrades in sea spray aerosol mimics, forming toxic photoproducts.

## Introduction

Personal care products are an emerging environmental concern due to their increased use and ecotoxicological impacts.^1^ Organic UV filters, particularly benzophenone-3 (BP3 or oxybenzone), are common in these products as well as in coatings and paints as a photoprotectant^2−4^ and prevalent in coastal environments^5−10^ due to direct application or through wastewater discharge.^8,11^ BP3 absorbs incoming UV radiation and dissipates energy nonradiatively through a photoinduced keto–enol tautomerization.^3,12^ This makes it an effective sunscreen and may contribute to its relatively long lifetime, which spans weeks to months in surface water.^13−15^

BP3’s extensive use and ubiquity in the marine environment raise concerns about its toxic impacts.^8,16,17^ Market research by Euromonitor reports a combined use of benzophenone class UV filters at 11.4 mg capita^–1^ day^–1^, amounting to 1386 t per annum in the U.S.^9^ Marine BP3 concentrations typically range between 100 ppt and 10 ppb,^8,9^ with levels as high as 1 ppm in crowded beach areas.^5^ In a National Academies review, the HC5 (hazardous concentration for 5% of species) of BP3 is calculated at 353 ppb for acute toxicity and 48.5 ppb for chronic toxicity.^9^ Additionally, BP3 is an endocrine disruptor^18,19^ and may contribute to developmental disorders.^20^

Airborne BP3 has been detected in gas and aerosol phases. Studies have reported varying concentrations of BP3 in urban and indoor air,^21,22^ with notable levels above wastewater treatment plants.^23^ BP3 can enter aerosols directly from sunscreen sprays^24^ or through evaporation and condensation processes.^21^ Selective transfer from the ocean surface into sea spray aerosols (SSA) through bubble rupturing and wave-breaking processes is another pathway by which hazardous organic and biological pollutants enter aerosols,^25−30^ given BP3’s hydrophobic nature (*K*_ow_ = 3.79^31^) and tendency to accumulate at the sea surface.^10^ Franklin et al.^32^ demonstrated that other organic UV filters (homosalate and octisalate) may transfer into artificial SSA generated in a wave channel using real collected seawater. While BP3 has been detected but not quantified yet in marine aerosol samples, BP3’s widespread presence in the ocean motivates this investigation into its photochemical transformations in seawater and SSA. Moreover, BP3’s photochemical lifetime in aerosols is unknown, which is critical for understanding its environmental fate.

Previous studies have shown that BP3 in seawater is resistant to direct photolysis but susceptible to indirect photolysis, with marine chromophoric matter playing a significant role.^33^ The environmental medium, e.g., solutions, thin films, or aerosols, and its physicochemical properties, such as viscosity, affect photochemical reaction pathways and rates.^34−38^ For example, octinoxate, another UV filter, degraded rapidly in thin films or suspended colloids but minimally in solution.^37^ Lignell et al.^39^ found that 2,4-dinitrophenol underwent faster photodegradation (by 1 order of magnitude) in a more viscous medium.^39^ Barsotti et al. found that photolysis quantum yields for other nitrophenols were the same in both aqueous (i.e., liquid) and more viscous media, suggesting that this phenomenon may be compound-specific.^40^

Aerosols^41−43^ and microdroplets^44^ exhibit accelerated reaction kinetics compared to bulk solutions. Studies have shown that smaller droplets and aerosols can concentrate organic constituents at the particle surface, where these compounds have incomplete solvation shells.^43,45^ This can enhance molecule-to-molecule interactions and inhibit the recombination of fragmented compounds, leading to faster degradation in aerosols.^43,44^ Furthermore, more ordered molecular orientations at the particle surface may sterically inhibit the vibrational relaxation of excited states.^37^

This study explores the role of the environmental medium (bulk solution and aerosol) and secondary constituents (NaCl and photosensitizers) in the photoinitiated degradation of oxybenzone. By systematically increasing the complexity of mixtures and lab-generated aerosols, from pure components to real seawater and lab-generated SSA mimics, we seek to understand the environmental fate of BP3 and its potential impact on particle toxicity.

## Materials and Methods

### Bulk Solution Experiments

Solutions of BP3 (TCI, 99%) and the model photosensitizer 4-benzoylbenzoic acid (4-BBA, Acros Organics, 99%) were prepared at ∼10 ppm (ppm) by weight in a 50:50 solvent mixture of methanol (MeOH, Fisher, 99.9%) and water (H_2_O; Milli-Q, 18 MΩ cm resistivity). This solvent mixture was chosen due to the insolubility of BP3 and 4-BBA in water. Methanol’s use as a cosolvent may lead to the scavenging of free radicals, such as hydroxyl radicals, which could potentially limit BP3’s indirect photosensitized decay.^46^ NaCl (Fisher, 99.5%) was added with a final solution mixing ratio of 35 parts per thousand for mixtures mimicking ocean water’s salinity. The pH was not controlled but measured at ∼6.1 for prepared seawater mimics.

Additionally, one set of experiments was conducted by adding 10 ppm of oxybenzone to real seawater collected from the end of Scripps Pier in La Jolla, California (32.867° N, 117.257° W) on September seventh, 2023. The nearby Southern California Coastal Ocean Observing System (SCCOOS) Automated Shore Station^47^ reported 10 μg L^–1^ of chlorophyll a (the threshold for a phytoplankton bloom),^48^ 8.2 mg L^–1^ of dissolved oxygen, 33.14‰ salinity, and a pH of 8.11 for this date. While the 10 ppm concentration of BP3 introduced into seawater is 10–1000x higher than concentrations reported in ocean water,^8^ this was done to match experimental concentrations and for detection purposes via UV–vis spectrophotometry.

Blanks containing the full solution matrix except for oxybenzone were prepared for each experiment. For example, this would mean solutions of BP3 in 50:50 MeOH:H_2_O would be paired with a blank of 50:50 MeOH:H_2_O, and solutions of BP3 + 4-BBA + NaCl in 50:50 MeOH:H_2_O would be paired with a blank solution of 4-BBA + NaCl in 50:50 MeOH:H_2_O.

The prepared solutions were placed in 10 mm path-length quartz cuvettes (Fireflysci type 21) sealed with a Teflon cap. The cuvettes were positioned on a magnetic stir plate and mixed with a stir bar operating at 300 rpm to ensure continuous mixing during each trial. Irradiation was achieved using an Air Mass 16S series solar simulator (Solar Light) calibrated to produce light intensity equivalent to 1 reference sun (760 W m^–2^), a proxy for the average daylight received over the contiguous United States. Light flux was measured by a model PMA2144 Digital Class II Pyranometer (Solar Light) at the beginning and end of each experiment. Minimal (<1%) drift was observed in spectral irradiance.

Photodegradation was monitored with a PerkinElmer Lambda 35 UV–vis spectrometer operating in dual beam mode, i.e., with automatic background subtraction. In this configuration, a blank cuvette was prepared consisting of the same solvent and cosolute mixture as the sample (i.e., all constituents of the sample except for BP3.) Measurements were taken at predetermined time intervals (0, 1, 5, 10, 15, 30, 60, and 120 min of light exposure) to monitor the evolution of the solutions over time. Since the solar simulator was tuned to the equivalence of 1 reference sun, these times are equivalent to the solar exposure time under clear skies at a zenith angle of 48.19°. Separately, for compositional analysis, samples of each mixture type were prepared at 100 ppm, and subsamples were exposed to solar-simulated light for 24 h to maximize photoproduct formation.

Cuvettes were also weighed with an analytical balance (Mettler Toledo model ME204) to measure any evaporation of the solvent (typically <1% throughout the experiments.) Subsequent spectra intensities were corrected to account for changes in the analyte concentration from solvent evaporation. The collected spectra were processed and analyzed using Spectragryph.^49^ Experimentally determined absorption spectra for each experimental solution type and further discussion can be seen in Figure S1 and the Supporting Information.

Samples were analyzed using a Vanquish UHPLC (Thermo Scientific) combined with Atmospheric Pressure Chemical Ionization (APCI) on the Orbitrap Elite Hybrid Linear Ion Trap-Orbitrap (Thermo Scientific). 20 μL of each sample was injected onto a Waters Symmetry C18 column (100 Å pore size, 5 μm particle size, 4.6 mm × 250 mm). The elution gradient consisted of pure HPLC-grade water (solvent A) and methanol (solvent B.) At a flow rate of 1 mL min^–1^, the gradient was as follows: 0 to 1.0 min at 70% B, 1.0 to 2.5 min transitioning from 70% to 5% B, 3.5 to 8.5 min transitioning from 5% to 85% B, followed by a 1.5 min equilibration phase at 70% B.

Following separation, samples were immediately analyzed using data-dependent acquisition in both positive and negative modes. APCI vaporizer temperature was set to 350 °C with a sheath, auxiliary, and sweep flow rate of 10, 30, and 5 arbitrary units, respectively. Discharge current was set to 5 μA with a capillary temperature of 300 °C and an S-lens RF level of 60.0. MS/MS scans were collected at a resolution of 60,000, collision energy of 35 kV, in an *m*/*z* range of 150.00–1500.00. Data were analyzed using Xcalibur (Thermo Scientific.)

### Aerosol Experiments

For experiments in the aerosol phase, solutions were atomized with a commercial constant output atomizer (TSI Inc. model 2076) and diluted to 5 L min^–1^ with zero air provided by a zero air generator (ZAG) (Sabio, model 1001). The solutions were prepared at 100 ppm by weight for each substituent in 100% MeOH. Note that this concentration is irrelevant to the ocean environment; it was chosen only to generate sufficient atomized aerosol mass. This solvent choice was necessary to ensure the complete dissolution of BP3 and 4-BBA, while low concentrations of NaCl are miscible in MeOH. The 100 ppm solution produced aerosol after atomization with mean geometric diameters between 90 and 140 nm (see Table S1 for more details.) For the spiked seawater trial, the seawater sample was first diluted to a salt concentration of 100 ppm, and BP3 dissolved in MeOH was added dropwise until a final BP3 concentration of 100 ppm. Aerosol size distributions were measured with a scanning electrical mobility sizer (Brechtel model 2100.) The differences and similarities of the atomized particles to real SSA is discussed in the Supporting Information.

Aerosol populations were subsequently introduced into an Aerodyne potential aerosol mass oxidative flow reactor (PAM-OFR).^50^ See Figure S2 for a flow diagram of our experimental setup. Inside the flow reactor, aerosol was exposed to wideband UV-B light provided by two lamps (LCD Lighting). Lamp spectral irradiance was between 300 and 350 nm, the highest energy range of incoming solar radiation at Earth’s surface. Output spectra can be found in Figure S3. Irradiance was controlled using a ballast at five different levels to the lamps.

Photon flux was measured directly by a TOCON GaP6 photodiode (sglux). It was also calculated by calibrating the lamps’ output photon flux via the photodegradation of NO_2_ as described by Lambe et al.^51^ (see Figure S4) and adjusted to the average NO_2_ absorptive cross-section between 300 and 350 nm. The photon flux was fitted to a 0-D box model run in KinSim^52^ (see Figure S5) and was determined to be between 8 × 10^14^ and 3 × 10^15^ photons cm^–2^ for the five different lamp settings. Using the standard reference solar spectrum at a 37° zenith angle, this photon flux between 300 and 350 nm is equivalent to 5–73% solar irradiance. The two methods showed good agreement, as shown in Figure S6. The photon flux from photodiode measurements was used for the remainder of this study.

Aerosol composition was measured with an extractive electrospray ionization time-of-flight (EESI-TOF) mass spectrometer (Aerodyne).^53^ This online (1 Hz) soft ionization approach allows for determining chemical formulas due to its high resolution, ∼5000 *m*/Δ*m* in the negative mode for *m*/*z* = 59.013853 (CH_3_COO^–^). Thus, it is particularly well suited to kinetics experiments of simple mixtures where the targeted analyte has no isomers.^38^

The EESI-TOF was operated in negative mode using a reagent solution of 2% (by weight) acetic acid, 48% H_2_O, and 50% acetonitrile spiked with 100 ppm of NaI for mass calibration with I^–^, I_2_^–^, and I_3_^–^. Using acetate as a reagent ion promotes the formation of [M – H] peaks via deprotonation.^38,86^ No iodide–analyte clusters, which may form in negative mode, were observed. Data was processed using Tofware (Aerodyne). Oxybenzone was detected as its [M – H]^−^ peak at *m*/*z* 227.

Photodegradation experiments under constant photon flux may be treated as pseudo-first-order reactions.^38^ Kinetic data was prepared by first drift-correcting and then normalizing signal intensities to *I* = 1 at *t* = 0 (see the Supporting Information for discussion on aerosol evaporation and drift correction). Taking the natural log of the normalized intensities for each exposure data point results in a linear decay plot where the slope represents the observed effective photolysis rate constant, *J*_eff,obs_ (as shown in eq 1):
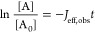
1This rate reflects the loss of BP3 from the sum of all photoinduced decay processes in the cuvette or the OFR. This includes both direct photolysis and photosensitized (or indirect) decay. No further analysis was performed to isolate the kinetics of direct or indirect photoinitiated decay.

*J*_eff,obs_ determined under experimental conditions was converted to an effective photolysis decay constant in the environment (*J*_eff,env_) by normalizing the output photon flux during experiments to the photon flux of standard spectral irradiance over the same wavelengths and multiplying by *J*_eff,obs_ as shown in eq 2 from Sareen et al.:^54^
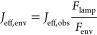
2This correction factor was 1 for all experiments in the bulk solution and ranged from 0.05 to 0.72 for experiments in the aerosol flow tube dependent on the different lamp voltages.

Aerosol samples for HPLC-MS/MS analysis were collected onto 90 mm quartz fiber filters inside a stainless-steel filter holder (Millipore). Samples were collected at the experimental flow rate of 3 L min^–1^ without an external pump. Flow rates were monitored by a flow meter (Siargo MF5700). Separate filters were used for aerosol collection in the dark, light, and blanks. The aerosol filter blanks followed EPA Method IO-5,^55^ which consisted of loading the filter holder, unloading, and storing the filters the same way as the samples. Immediately after collection, the filter was submerged in ∼15 mL of acetonitrile (Fisher, 99.9%) inside a cleaned and baked Pyrex scintillation vial and sonicated for 30 min in an ultrasonicated bath (Branson 3800). Since water was not used during extraction, our analysis may not include all water-soluble components in the particles. This solution was then dried via rotary evaporation (Buchi R-100) and reconstituted in 1 mL of acetonitrile for analysis using the same HP-LCMS/MS procedure described earlier.

### Modeling of Electronic Structures and Toxicity

The nuclei positions of ground state BP3 and its degradation products were constructed using the Avogadro 4.2 molecular visualization program on a Windows platform. The quantum chemistry package ORCA (version 5.0.3) performed all computations.^56,57^ The initial geometry optimizations were performed using DFT with B3LYP functional with def2-TZVP basis set, and bond angle constraints were applied when appropriate. Coulomb integrals and COSX numerical integration for HF exchange were adopted for the RI-J approximation. The geometry was further optimized using ethanol as an implicit solvent. Finally, a coupled cluster technique, STEOM-DLPNO-CCSD,^58^ was employed to calculate the energy of the electronic transitions of the molecules.

Separately, the geometry of a BP3 molecule was optimized using ωB87x-D3 with the def2-TZVP basis set. Its deprotonated and protonated counterparts were subsequently computed using the same geometry to perform the analysis of the Fukui function. The representations were generated using the VESTA^59^ visualization package.

Compound toxicity was estimated by calculating the rat oral LD_50_ of BP3 and its detected products using EPA’s Toxicity Estimation Software Tool (TEST),^60^ a quantitative structure–activity relationship model that predicts toxicological end points based on molecular descriptors of experimental data. When experimental data is available within TEST, as for oxybenzone and benzophenone, this value was preferentially used over simulated LD_50_ values. This work used the prediction algorithm for oral rat LD50, based on a training set of over 7400 chemicals. This toxicity end point typically exhibits the highest degree of experimental error compared to aqueous toxicity. To reduce error, the consensus (average) toxicity predicted by both hierarchical and nearest-neighbor approaches was used to estimate toxicity.

## Results and Discussion

### Differences in the Photoinitiated Decay Kinetics of Pure-Component BP3 between Bulk Solutions and Aerosols

**Figure 1:**
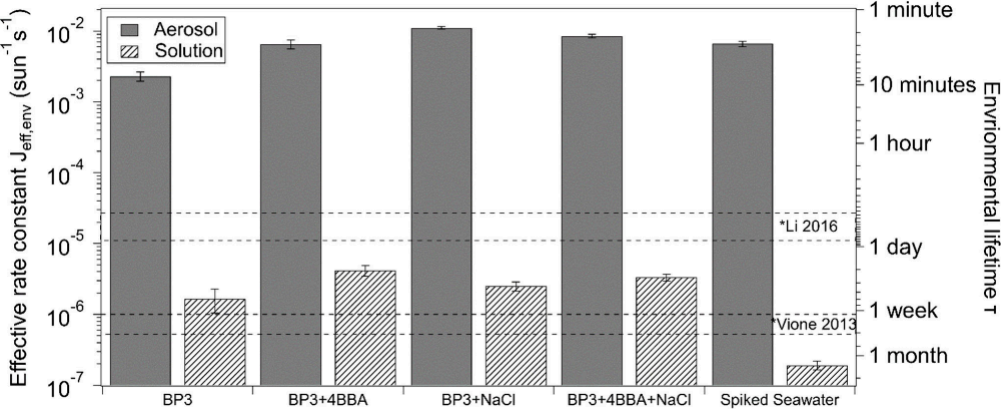
Effective photolytic rate constants and estimated chemical lifetimes of BP3 upon exposure to solar irradiation in the aerosol and bulk solution phases for the different mixtures. Previous studies by Li et al.^33^ and Vione et al.^14^ are shown for comparison. The range for Li et al. is between real seawater (upper) and freshwater (lower). The range for Vione et al. is between low concentrations of nitrate and carbonate (upper) and high concentrations (lower).

We observed profound differences in photoinduced decay between the bulk solution and aerosol phase experiments, as shown in Figure 1. BP3 exhibited slow decay, decreasing in concentration by 5% or less over hours in the bulk solution while undergoing rapid degradation, decreasing in concentration by up to 60% in the aerosol phase on the order of minutes of exposure time, varying based on the mixture (see average decay curves in Figure S7).

Employing a pseudo-first-order kinetics treatment (see Methods) to the average degradation observed during each experiment, we calculated effective photolysis decay constants, *J*_eff,env_, and environmental lifetime, τ, plotted in Figure 1 and listed in Table S2. The effective BP3 photodegradation rates of pure-component BP3 in bulk solution were ∼2 × 10^–6^ s^–1^ compared to ∼2 × 10^–3^ s^–1^ in aerosols. These are equivalent to τ ≈ 6 days and 8 min, respectively.

The relatively slow photoinduced decay in solution was expected. Most studies show no significant degradation of oxybenzone in solution to solar-simulated light for experimental times up to 24 h.^13,15,61^ Vione et al. reported photolysis of oxybenzone in freshwater with an environmental lifetime of 9–15 days, depending on carbonate and nitrate concentrations.^14^ In contrast, Li et al. 2016 reported an environmental lifetime of 9 h in freshwater and 20 h in seawater.^33^

**Figure 2:**
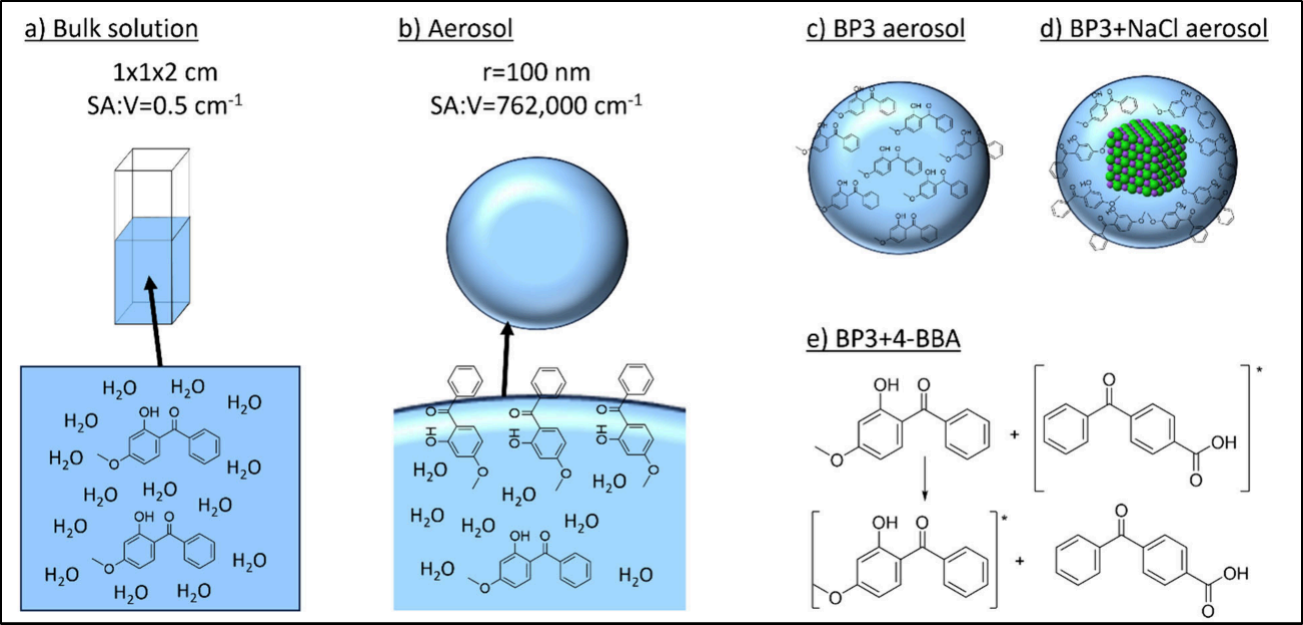
Illustration of the different factors impacting BP3 loss in solutions and aerosols. (a) BP3 solution in a cuvette with a full solvent cage. Dimensions of the cuvette, radius of the aerosol, and calculated surface area to volume ratios (SA:V) are shown. (b) BP3 aerosol with incomplete solvation at the air-particle interface. (c) BP3 aerosol with BP3 distributed through the bulk and interface. (d) BP3 + NaCl aerosol with BP3 driven to the surface. (e) Type I photosensitization scheme of excited state transfer between 4-BBA and BP3.

We ascribe the enhanced degradation in aerosols primarily to the much larger surface area-to-volume ratio (see Figure 2a,b). This leads to more BP3 molecules at the particle-air interface than the bulk, where incomplete solvation may greatly enhance photochemical reactivity.^41,43,44^ Solvation can often slow reactions in solutions compared to the gas phase. The partial solvation at the air-particle interface often exhibits rates between fast gas-phase and slow solution-phase reactions.^44^

Additionally, organic-containing aerosols may exhibit higher viscosities akin to semisolids and solids.^62^ More viscous environments limit the rotational freedom of excited states’ rotations,^63,64^ which BP3 relies on to relax vibrationally. Thus, a higher-viscosity molecular environment would prolong BP3’s excited state, allowing it greater opportunity to decay rather than relax.

### BP3 + NaCl Binary Mixtures

In bulk solutions, there was a minor but statistically insignificant (*p* > 0.05) increase in BP3 degradation for the BP3 + NaCl binary system compared to BP3 alone. Berembeim et al. found that the presence of Na^+^ can interfere with BP3’s ability to relax via keto–enol tautomerization by disrupting its intramolecular hydrogen bond.^65^ This would extend the lifetime of BP3’s excited state and promote its decay instead.

In contrast to the bulk solution, adding NaCl in the aerosol phase led to the greatest increase (4.5×) in rate compared to pure BP3. As shown in Figure S8, the particles mixed with NaCl exhibited visual characteristics of phase-separated and partially engulfed particles with a possible NaCl core and BP3 coating. These are similar morphologies observed in other inorganic salt-organic carbon mixtures.^66^ As illustrated in Figure 2c,d, this separation of components leads to a population of BP3 molecules preferentially occupying the particle’s surface,^67^ where this more viscous and less solvated molecular environment could lead to enhanced degradation,^41,43,44^ suggesting the aerosol particle’s morphology could significantly influence BP3’s photoinduced degradation.

### BP3 + 4-BBA Binary Mixtures

In the bulk solution, there was a significant (*p* < 0.01) increase (2×) in the rate constant calculated for the BP3 + 4-BBA binary system compared to BP3. This confirms that 4-BBA acts as a photosensitizer in increasing the photodegradation rate of BP3, either through direct triplet state energy transfer (Type 1, Figure 2e) or through the formation of reactive oxygen species (Type 2).^68^ While we did not perform oxidant quenching experiments to determine differences between Type 1 and Type 2 initiated processes, Vione et al. 2013 found that both forms of photosensitization occur in freshwater systems, with Type 1 prevailing under high dissolved organic matter concentrations (where there would be more photosensitizer-BP3 interactions) and Type 2 prevailing under low photosensitizer concentrations (where there would be less scavenging of OH by organics other than BP3).^14^

In the aerosol phase, the addition of 4-BBA to BP3 also significantly (*p* < 0.01) increased (2.5×) rates of degradation. We expect similar mechanisms of photodegradation to occur in the aerosol phase as in the bulk solution. However, the larger increase in the aerosol phase may be explained by the closer proximity and incomplete solvation of organic molecules in the condensed particle phase compared to the bulk solution leading to more BP3–4-BBA* interactions.^41,43^

### BP3 + NaCl + 4-BBA Ternary Mixtures

In the bulk solution, adding NaCl to the BP3 + 4-BBA binary system led to an insignificant (*p* > 0.05) but a minor decrease in reaction rate compared to BP3 + 4-BBA but a significant (*p* < 0.05) increase compared to BP3 + NaCl. We hypothesize that this could be due to the presence of NaCl leading to vibrational collisions between NaCl and ^3^4-BBA*, which may quench the triplet state that may have otherwise reacted with BP3. This effect has been seen in other mixtures of halides and photosensitizing molecules, e.g., NaCl and 4-BBA in aerosols^38^ and sea salt halides mixed with imidazole-2-carboxaldehyde in bulk solution.^69^

In the aerosol phase, a similar modulating effect was observed where the ternary mixture was faster than one binary mixture (BP3 + 4BBA, insignificant, *p* > 0.05) and slower than the other binary mixture (BP3 + NaCl, *p* < 0.05). One reason for the decreased rate compared to the BP3 + NaCl aerosol is that 4-BBA may competitively occupy the surface of the aerosol, driving more of the BP3 into the bulk of the aerosol compared to the binary mixture. Although including 4-BBA would introduce additional photosensitized decay pathways, this appears less impactful than the surface enrichment caused by NaCl.

Additionally, the quenching of 4-BBA by Cl^–^ may lead to the formation of reactive chlorine species either directly or through ·OH-mediated oxidation pathways, which have been shown to contribute to the UV-initiated removal of BP3.^70−72^ This may be an additional pathway leading to enhanced degradation in the bulk solution compared to the BP3 + NaCl binary mixture, but it does not appear to fully compensate for the inhibitory effects of 4-BBA in the ternary aerosol compared to the BP3 + NaCl binary aerosol.

### Photoinduced Decay of BP3 in Real Seawater

For the bulk solution experiment conducted by spiking BP3 into real seawater, we observed no degradation and instead observed a steady enhancement (up to 8%) for the first 3 days of irradiation. We ascribe this to the general insolubility of BP3 in seawater and expect it first to be adsorbed to particulate matter in the seawater. Light exposure can lead to the dissolution of organic matter from particulate matter,^73^ explaining its enhancement over time. However, after this enhancement period, BP3 decreased steadily, and we used this period to calculate its photoinduced decay. We caution that this may not account for particulate adsorption and desorption and instead reflects the decay after a steady state was reached.

The resulting decay (with an environmental lifetime of over 1 month) is significantly slower than the experimental mixtures (in days) and the decay observed by Li et al. in real seawater (with an environmental lifetime of 20 h).^33^ However, other studies simply state that BP3 persists in ocean water without reporting any degradation.^13,61^

Another reason for the slower observed kinetics in the seawater solution may be the difference in the protonation state of BP3. The seawater experiment was conducted at a final pH of 7.1 compared to 6.0 in the other solutions. Because BP3 has an acid dissociation constant (p*K*_a_ = 7.1), a higher pH leads to more deprotonated BP3 in the solution. The impacts of the protonation state on BP3’s photodegradation are inconclusive. However, a study by Wong et al., which investigated the gas-phase photodegradation of protonated, neutral, and deprotonated BP3, found that absorption by the deprotonated form of BP3 was significantly different from its neutral form and displayed photodissociation patterns indicative of longer-lived excited states.^12^ Similarly, Li et al., who studied BP3 in real seawater, found that only the deprotonated form was susceptible to direct photodegradation.^33^ Oceanic pH is typically 8.0, a limitation of this study. Thus, the bulk solution experiments primarily serve to inform chemical mechanisms and intercomparisons between environmental phases rather than serving as a direct representation of the ocean.

Additionally, real seawater contains a more complex dissolved organic matter system than the 4-BBA used in experimental mixtures. This may screen incoming light, reducing direct photolysis and quench any excited photosensitizers or reactive oxygen species produced, reducing photosensitization efficiency.^14^ Trueblood et al. found that the photosensitized degradation of nonanoic acid was slower in the presence of an extracted marine DOM sample than with 4-BBA and humic acid. They explain this as resulting from the high abundance of carboxylic-rich alicyclic molecules, which are less photoactive than aromatic photosensitizers.^36^

The experiments conducted by spiking oxybenzone into seawater followed by atomization exhibited an insignificant (*p* > 0.05) decrease compared to the ternary mixture. This could mean that the components atomized in seawater have similar properties and effects on BP3 photodegradation as the ternary mixture. While this result suggests the photoinitiated decay of BP3 in SSA may be as fast as those from other mixed aerosols in this study, it is important to note that the components transferred into SSA produced from the natural process of wave breaking and bubble rupturing at the sea surface are different from those that get atomized.^74^ Only dissolved materials get atomized, whereas nondissolved components, like lipids, surfactants, and particulate organic carbon, at the sea surface can transfer into SSA.^75^ These components modulate SSA’s physicochemical properties, surface tension, and mixing states, potentially different from the particles generated from atomized seawater. Therefore, this analysis does not say that the same degradation rates would be observed in real SSA; instead, it shows no significant inhibition of BP3 degradation as observed in the bulk seawater solution.

### Identified Transformation Products

**Figure 3:**
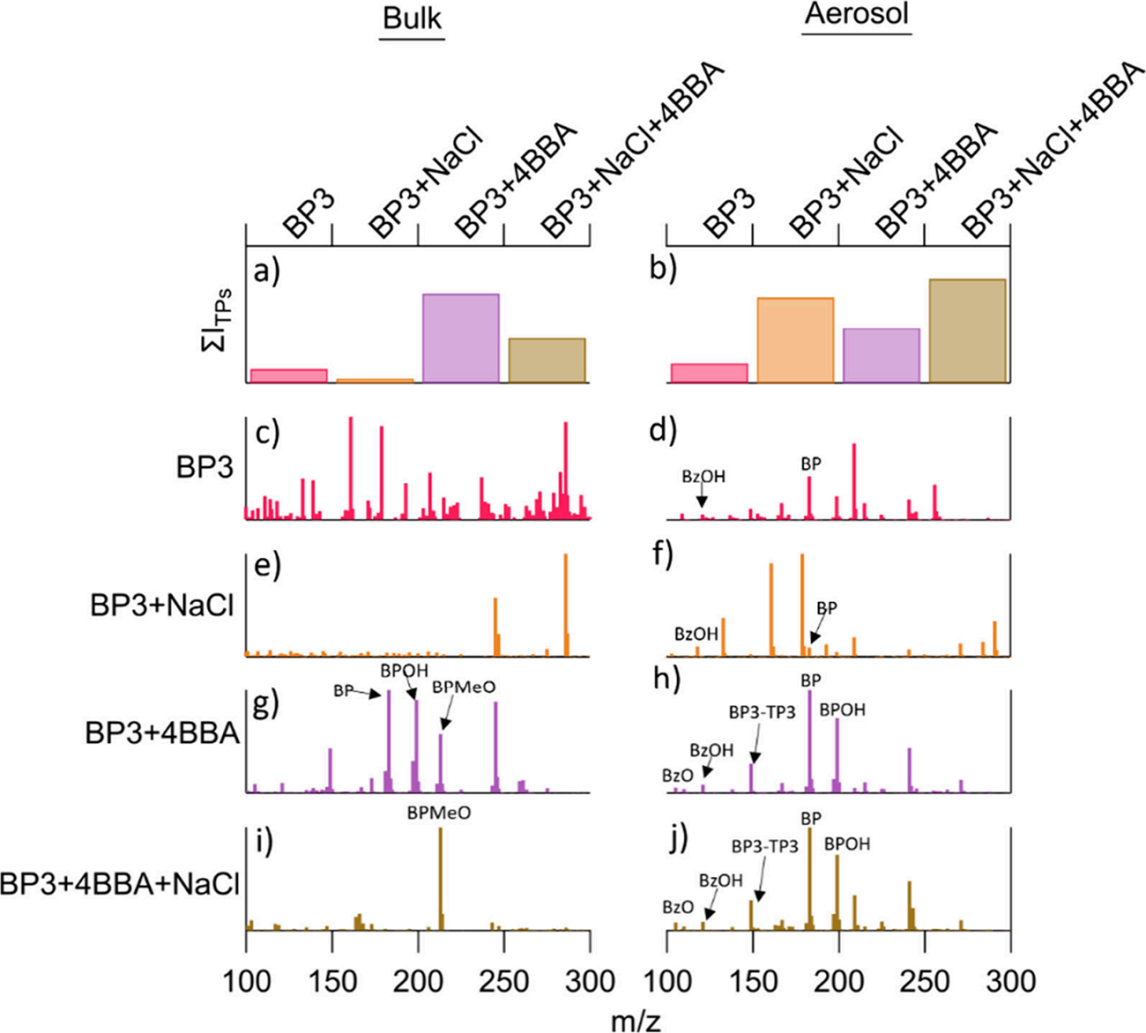
(a) Summed ion intensities of the detected transformation products in bulk solutions based on mixture type. (b) Same as (a) but for the aerosol phase. For comparison, the intensity of the bulk solution transformation products is multiplied by 30. (c–j) Phototransformation product mass spectra for the different indicated mixtures in bulk solutions (left) and aerosols (right).

Compositional analysis via HPLC MS/MS was conducted in a separate set of experiments where all bulk solutions were irradiated continuously for 24 h in the cuvettes by the solar simulator and aerosols for 8 min of equivalent solar exposure by the UV–B lamps inside the OFR. The resulting mass spectra of samples before irradiation (i.e., in the dark) were subtracted from mass spectra after irradiation, plotted in Figure 3. The compounds identified in the mass spectra that were significantly above the background (i.e., 3 standard deviations above the background for at least one sample type) are listed in Table 1. MS2 spectra of each identified BP3 product are shown in Figure S9.

**Table 1:** Detected Compound IDs, MS1 Parent Ions, and Major MS2 Fragmentation Ions

compound ID	parent ion	major fragmentation ions
BP3	229 (M + H)^+^	105, 151
BP3-TP1 (benzaldehyde, BzO)	106 (M – H)^+^	N/A
BP3-TP2 (benzoic acid, BzOH)	122 (M – H)^+^	N/A
BP3-TP3 (2-hydroxy-4-methoxybenzaldehyde)	153 (M + H)^+^	109, 135
BP3-TP4 (benzophenone, BP)	183 (M + H)^+^	105
BP3-TP5 (2-hydroxybenzophenone, BPOH)	199 (M + H)^+^	105, 121
BP3-TP6 (4-methoxybenzophenone, BPMeO)	213 (M + H)^+^	153, 181, 198
BP3-TP7 (2,4-dimethoxybenzophenone)	241 (M – H)^+^	105, 163
4-BBA (4-benzoylbenzoic acid)	227 (M + H)^+^	105, 149
4-BBA-TP1 (4-benzoylbenzyl alcohol)	213	105, 135, 183

Without quantification of each transformation product, we cannot confidently compare the branching ratios between different organic species but can compare the formation of each compound between different mixture types. Almost all transformation products (TPs) exhibited the highest production in the BP3 + 4-BBA binary mixture in the bulk solution. This is to be expected, as these experiments showed the greatest degradation of BP3. Indeed, the order of TP production was completely in line with the rates of photoinitiated decay.

Interestingly, this trend was not observed in the aerosol phase. Instead, the ternary aerosol showed more TP production than BP3 + NaCl, which exhibited the greatest decay. This may be due to additional TPs from the transformations of 4-BBA in this aerosol sample. In subsequent sections, we will deconvolute the contributions of BP3 from those of 4-BBA.

Previous work by Vione et al. identified major photoproducts of BP3 photolysis in the bulk solution to be benzoic acid and benzaldehyde resulting from the rupture of the carbon–carbon bond linking the carbonyl group to the aromatic ring containing the methoxy and hydroxyl moieties.^14^ Wong et al. observed rupture of this as well as the opposite.^12^

We observed the formation of benzaldehyde transformation product (BP3-TP1, BzO) in all experiments conducted in the bulk solution phase and aerosol experiments containing NaCl. In contrast, benzoic acid (BP3-TP2, BzOH) was a minor product (<1000 ions) in all bulk experiments and was detected in aerosol experiments not containing NaCl. As observed by Wong et al., a substituted benzaldehyde with hydroxyl and methoxy moieties (BP-TP3) would form from the rupture of the carbon–carbon bond between the carbonyl group and the unsubstituted aromatic ring.^12^ This product was only observed in bulk experiments in binary or ternary mixtures containing 4-BBA, suggesting that the C–C bond connecting the substituted ring and the central ketone is more prone to cleavage than the C–C bond in the unsubstituted ring.

Additional transformation products were identified by their MS2 spectra as less substituted compounds resulting from the removal of the hydroxyl and/or methoxy groups from oxybenzone. Notably, removing both constituents results in the formation of benzophenone, a known carcinogen.^76^ Benzophenone (BP), 2-hydroxybenzophenone (BPOH), and 4-methoxybenzophenone (BPMeO) were all observed.

In the bulk solution, the production of BPMeO was observed in both the binary BP3 + 4-BBA trial and the ternary mixture. BP was only measured in the BP3 + 4-BBA experiment. In the aerosol phase, all experiments led to the production of BPOH and the formation of BP. To our knowledge, this is the first study reporting this sequence of degradation products from BP3.

The transformation products detected at the greatest intensity in aerosol samples were the derivatives produced by removing either the hydroxyl or methoxy group from BP3 and removing both to form benzophenone. Because these products are formed in pure BP3 aerosols, we expect them to result from direct photolysis, which results in the lysing of these C–O bonds.

As BP, BPOH, and BPMeO remain the dominant TPs, we expect these degradative pathways to be enhanced by Type 1 photosensitizing reactions with ^3^4-BBA*, which behave similarly to photon absorption. We did not observe direct evidence of Type 2 photosensitizing reactions involving reactive oxygen species, which would generally result in more functionalized products. The cosolvent methanol may partially influence this in the bulk solution, which could slow BP3 decay by scavenging reactive oxygen species. However, no evidence of Type 2 reactions was observed in systems without methanol, including the bulk seawater and aerosol samples. This suggests reactive oxygen species were not efficiently generated under the experimental conditions or were too short-lived to react appreciably with BP3.

While we could not confidently determine every transformation product observed, the relative production levels provide additional confirmation of the different rates of photodegradation of different mixtures. Notably, trials containing 4-BBA exhibited the production of products with integrated signal intensities an order of magnitude greater than other bulk experiments (Figure 3a).

As both BP3 and 4-BBA are benzophenone derivatives, care was made to deconvolute similar transformation products. To identify transformation products of 4-BBA, experiments with only the photosensitizer were run. In the bulk solution, the primary TP (*m*/*z* of 213) was tentatively identified as 4-benzoylbenzyl alcohol formed from the reduction of 4-BBA’s carboxylic acid group (see Figure S10). It is important to note that 4-benzoylbenzyl alcohol shares a chemical formula and thus *m*/*z* with BPMeO and was deconvoluted via LC and identified based on their MS2 spectra. Grzyb et al.^77^ observed the formation of 4-benzoyl methyl aldehyde was observed following 4-BBA’s use as a photosensitizer, showing precedent for the reduction of 4-BBA while acting as a photosensitizer.

### Computational Investigation of Photoinitiated Reactive Sites

We employed a computational frontier molecular orbital model to investigate the observed cleavage of C–C and C–O bonds. This approach assumes that the breakage of a bond originates from the excitation of an electron from the highest occupied molecular orbital (HOMO) and that the formation of a bond is the consequence of an acceptance of an electron from a donor to the lowest unoccupied molecular orbital (LUMO). Janak’s theorem further suggests that HOMO and LUMO could serve as proxies to understand the ionization potential and electron affinity, respectively, of the molecule. In other words, by studying the HOMO and the LUMO of a molecule, we can provide an additional layer of insight if the proposed mechanism of a reaction is reasonable.

**Figure 4:**
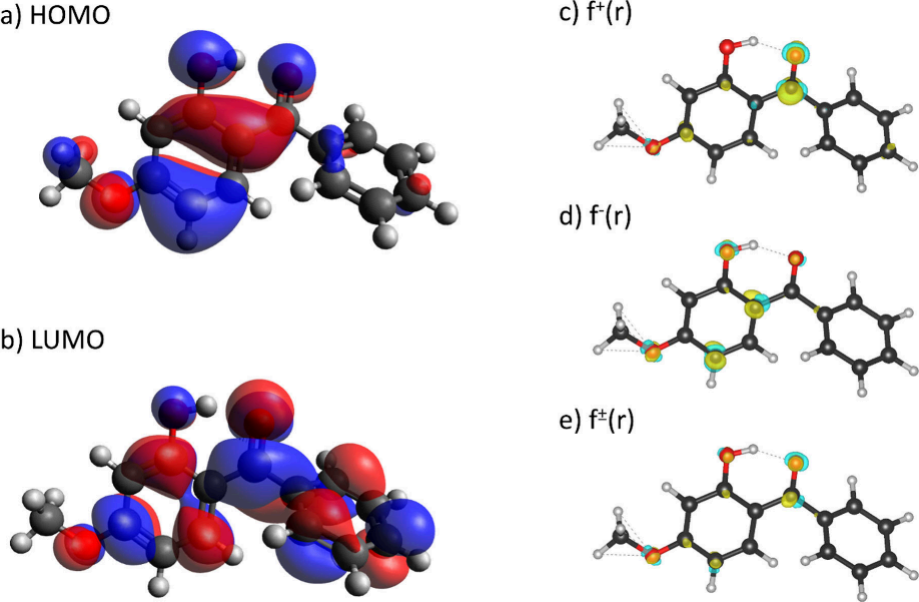
Graphic representations of BP3’s (a) HOMO, (b) LUMO, (c) cationic Fukui function, (d) anionic Fukui function, and (e) radical Fukui function.

A model of BP3’s HOMO (see Figure 4a) shows a prominent orbital lobe over the C–C connecting the substituted ring (carbonyl-α carbon bond) with a mix of both n and pi-character. An available electron may be promoted to an excited state via an electromagnetic wave with an energy higher or equal to its HOMO–LUMO gap, which is 3.92 eV, or 316 nm; the electron may relax back to the ground state or may overcome the ionization potential and dissociates, providing a pathway to photodegradation. The LUMO of BP3 (see Figure 4b) exhibits a dominant π* character across both benzene moieties. This may explain the observed production of both benzaldehyde and 2-hydroxy-4-methoxybenzaldehyde upon photolysis: carbonyl-α carbon bond breakage of either side of the two rings may be the result of an intermediate species or transition state accepting a relaxing electron to either one of the benzene moieties which will destabilize its opposing C–C bond.

The above analysis can be further investigated in conjunction with the Fukui function.^78^ In short, the Fukui function takes advantage of the approach of DFT to look for changes in electron density in frontier orbitals induced by changes in the number of electrons at a specific geometry and the molecule’s potential. The Fukui functions are described below:
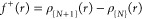
3
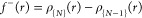
4
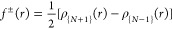
5where ρ is the electron density and *N* is the number of electrons. For a neutrally charged molecule with *N* electrons, *N* + 1 will be its anionic counterpart and *N* – 1 will be cationic. Hence, eq 3 is related to reactivity toward nucleophiles and eq 4 to reactivity toward electrophiles; eq 5 provides information on reactivity in the case of radical attack.

As shown in Figure 4c, the electrophilic sites of BP3 are located almost exclusively on the carbonyl carbon, attracting nucleophiles. The tertiary structure of the carbon allows lone pair electrons or unpaired electrons to be stabilized and promotes further reactions after photoirradiation, such as the cleavage of the α–β carbon bond discussed earlier.

The nucleophilic sites (see Figure 4d) of BP3 are spread across the more substituted aromatic ring of the two. In particular, the oxygen on the hydroxyl and the methoxy groups are susceptible to protonation, which may promote their stability as leaving groups, leading to the observed formation of BPOH and BPMeO. We note that the increase in reactivity of the two β-carbons of the two functional groups is counteracted by their participation in the aromatic ring.

The Fukui function for radical attack is a straightforward pictorial prediction of the fate of the BP3 upon photoirradiation (see Figure 4e). It shows prominence in electron density differences on all reactive sites leading to each observed transformation product.

### Proposed Mechanism of Photoinduced Decay and Effects of Medium and Secondary Constituents

**Figure 5:**
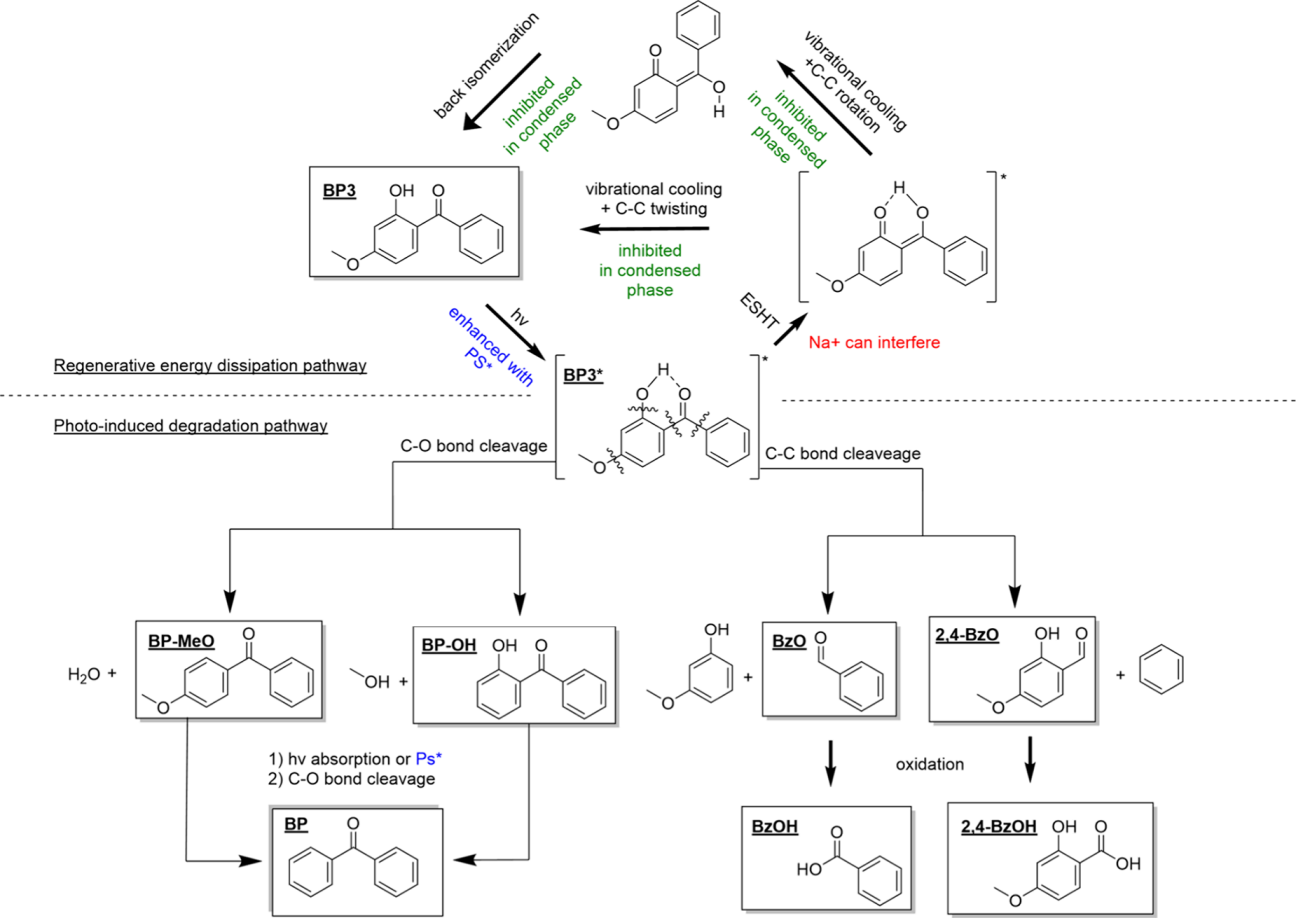
Transformation pathways of BP3 either regenerate ground-state BP3 or undergo photoinduced degradation. Detected compounds are shown in boxes and labeled according to their abbreviations listed in Table 1. The effects of the medium are shown in green, the effects of Na^+^ are shown in red, and the effects of photosensitizers are shown in blue.

Upon exposure to UV light, BP3 can regeneratively dissipate its energy either directly or through an excited state hydrogen transfer (ESHT) followed by a keto–enol tautomerization paired with molecular rotation.^3^ If this process does not occur, it may instead undergo photoinitiated degradation.^14^Figure 5 illustrates both pathways. Physical and chemical changes that promote or inhibit these pathways will impact the photoinduced degradation kinetics observed. Here, we separate the different impacts of the environmental medium and the presence of salts and photosensitizers and quantify their impacts on environmental lifetime.

As explained earlier, the impact of environmental medium is predominantly concerned with the concentration of the population of BP3 at the surface and generally increased viscosity in organic aerosol layers. BP3 at the surface is less well-solvated and thus has less of a hydrogen bonding network to facilitate its ESHT.^45^ Additionally, the increased molecular order at the interface^45^ and higher viscosity of organic-rich aerosol^62^ may inhibit the molecular rotations needed to release its excited state energy and back isomerize into BP3.

The introduction of salt led to a small increase in photoinitiated decay in solution and the largest increase in aerosol. Na^+^ may directly interfere with the ESHT by occupying the space between the hydroxyl and ketone groups on BP3.^65^ Additionally, the presence of NaCl, especially in the moderate relative humidity in this study, may drive the population of BP3 further to the surface, also known as “salting-out”, due to reduced organic solubility,^79,80^ amplifying the effects of interfacial reactivity.

Introducing the photosensitizer 4-BBA led to the apparent enhancement of photoinduced degradation through direct energy transfer (Type 1) and potentially the production of reactive oxygen species (Type 2).^35,36,68^ Our study did not observe direct evidence of any Type 2 reactions. As both BP3 and 4-BBA absorb UV radiation, they may also screen incoming light from BP3, reducing its direct photolysis. This did not seem to outcompete the effectiveness 4-BBA has as a photosensitizer except for in the ternary aerosol, where the addition of 4-BBA to BP3 + NaCl aerosol led to a small but significant (*p* < 0.05) decrease in reactivity.

### Environmental Implications

**Figure 6:**
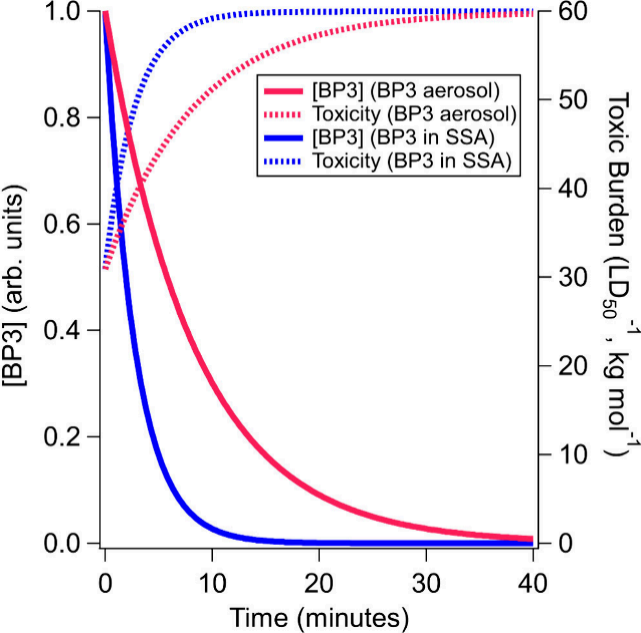
Estimated toxic burden over time for BP3 in the aerosol phase for pure BP3 aerosol (pink) and BP3 in a SSA mimic (blue). Experimental values were used for BP3, and the average of detected BP3 photoproducts was used to estimate product toxicity.

Notably, every transformation product identified in this study exhibits higher experimental and/or predicted toxicity than BP3 (see Table S3). This shows that the toxic loading of BP3 in the environment may not be reduced via photolysis, at least through its first few generation products. Figure 6 shows a representation of the aerosol toxic burden, calculated as the inverse of the estimated LD_50_, doubling over 40 min in the atmosphere. Differences between pure BP3 (in pink) and BP3 in the SSA mimic (in blue) are most pronounced within this short time scale, which may be relevant to the SSA locally generated and inhaled at the coast. In the real atmosphere, this may be representative of BP3 aerosol produced by a spray sunscreen vs BP3 as an organic component of SSA.

These results suggest that BP3 may be under-detected in environmental assays due to its rapid phototransformations in the aerosol phase to a diversity of transformation products. This is likely the case for other UV-absorbing compounds.

This work identified the first two generations of transformation products, but subsequent reactions may occur over the lifetime of aerosols in the atmosphere (up to weeks). Generally, fragmented photoproducts shift their absorption further into the UV range, making them more resistant to photolysis at tropospherically relevant wavelengths via photobleaching.^81^ However, reactive atmospheric trace gases, such as Cl, ·OH, and O_3_, may also oxidize BP3 and its photoproducts.^38,82,83^ The hydroxyl radical is often referred to as the “detergent of the atmosphere”,^84^ as it is the major removal pathway for many organic compounds. The EPA AOPWIN model estimates reactivity with ·OH based on structural characteristics.^85^Table S3 lists estimated atmospheric lifetimes (which the model conducts in the gas phase). This reveals that some TPs, such as benzoic acid, may be very long-lived compared to ·OH, with an estimated atmospheric lifetime of 9 days. Others, like 2-hydroxy-4-methoxy benzoic acid, have estimated lifetimes as short as 0.9 h. Thus, a more comprehensive environmental lifecycle analysis of BP3 is necessary to fully constrain the toxic burden of BP3 and its photoproducts in the atmosphere.

These results demonstrate the importance of the marine matrix when determining the environmental lifetime and fate of oxybenzone in seawater and SSA. In particular, the competing influences of photosensitizing organic molecules and NaCl modulate the environmental photoinitiated degradation of this compound. The aerosol phase promotes significantly faster photoinduced decay of BP3 compared to bulk solutions, likely due to differences in surface activity and viscosity. The role of photosensitizing material is most pronounced in the bulk solution, and NaCl is the most influential in aerosols. As noted before, for experimental reasons, the bulk solution experiments used BP3 concentrations greater than those in the environment. While the measured loss rates of BP3 in solution were similar in magnitude to other studies presented in Figure 1, the greater concentrations may lead to different loss rates than in natural marine aquatic environments.

Furthermore, methanol, due to its high concentration in the bulk solutions, could have led to some suppression in the decay rates of BP3 by scavenging reactive oxygen species but this is not unlike the surface microlayer of oceans (where BP3 accumulates), which is characterized by a high concentration of organic matter. Therefore, we deduce that similar scavenging will occur by myriad organic carbon molecules, potentially limiting BP3 decay by Type 2 reactions in the real sea surface microlayer.

Organic UV filters such as BP3 are common and increasingly found in all environmental mediums. These results suggest that BP3 may persist in the ocean but, upon aerosolization, is prone to rapid photoinduced decay, which produces more toxic compounds. Further studies on the ecotoxicological impacts of its transformation products should be performed to fully constrain this compound’s environmental and public health impacts.

## Supporting Information Available

UV–vis spectra, schematic of setup, lamp output spectrum, photolysis rate calibrations, aerosol and bulk solution decay curves, particle microscopy images, fragmentation mass spectra, mass losses from evaporation, and tabulated results and experimental details including solution pH, experimental relative humidity, temperature, and surface-area-weighted particle diameters (PDF)
