# Supplementary material for: Photoinitiated Degradation Kinetics of the Organic UV Filter Oxybenzone in Solutions and Aerosols: Impacts of Salt, Photosensitizers, and the Medium
Source: ACS EST Air. 2024 Oct 16;1(11):1430–41. doi: 10.1021/acsestair.4c00149 (PMC11555681; doi:10.1021/acsestair.4c00149)
Supplement: Supplementary file 1 — ea4c00149_si_001.pdf [file ea4c00149_si_001.pdf]

Supporting information for:

Photo-initiated degradation kinetics of the organic UV filter oxybenzone in solutions and aerosols: impacts of salt, photosensitizers, and the medium

Adam Cooper[1], Alexis Shenkiryk[2], Henry Chin[1], Maya Morris[1], Lincoln Mehndiratta[1], Kanuri Roundtree[1], Tessa Tafuri[1], and Jonathan H. Slade[1]*

[1]*Department of Chemistry & Biochemistry, University of California, San Diego, La Jolla, CA 92093, USA*

[2]*Department of Chemistry & Biochemistry, University of California, Los Angeles, Los Angeles, CA, 90095, USA*

**To whom correspondence should be addressed: jhslade@ucsd.edu*

Contents:

19 figures (Figures S1 to S19)

3 tables (Tables S1 to S3)

1 text (Text S1)

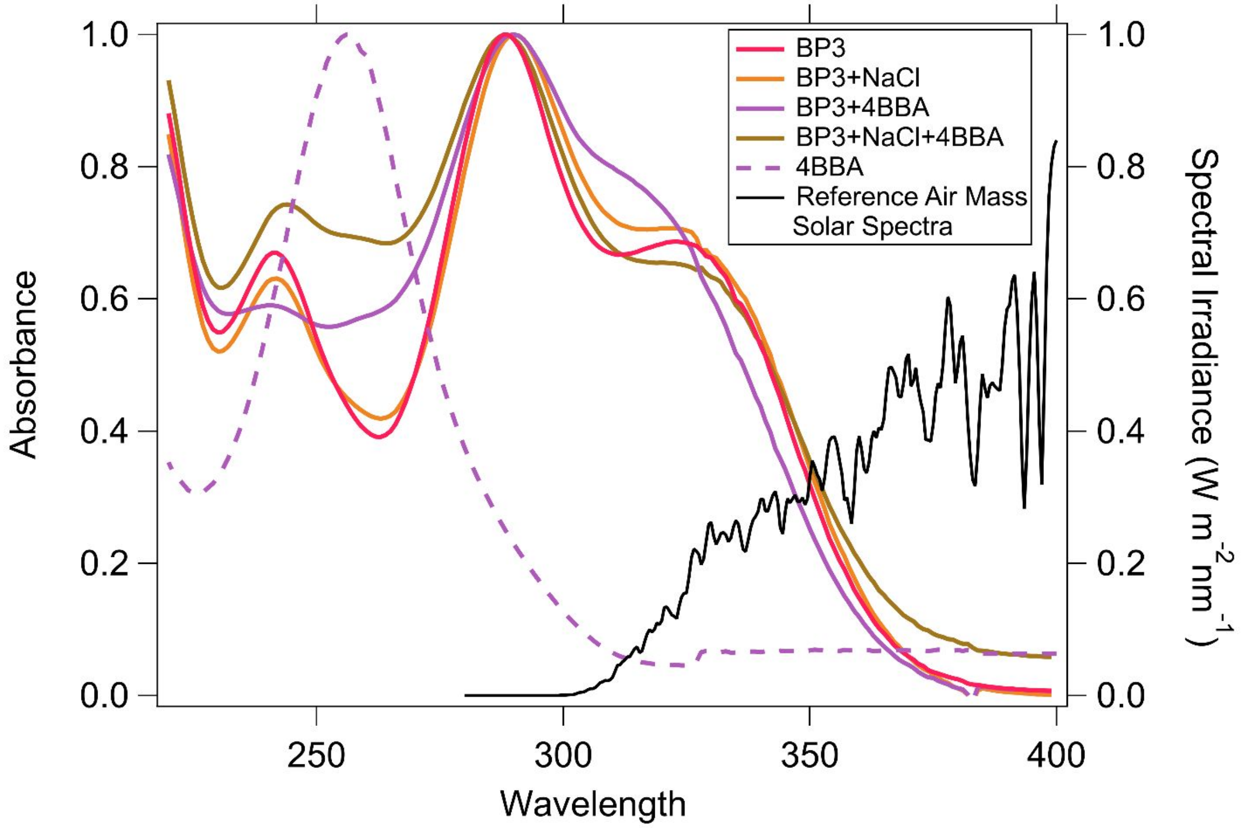

Figure S1. UV Vis absorption spectra of the pure, binary, and ternary solutions prior to irradiation normalized to λ_{max}=1. On the right axis, spectral irradiance of the standard solar spectra is shown for atmospheric reference.

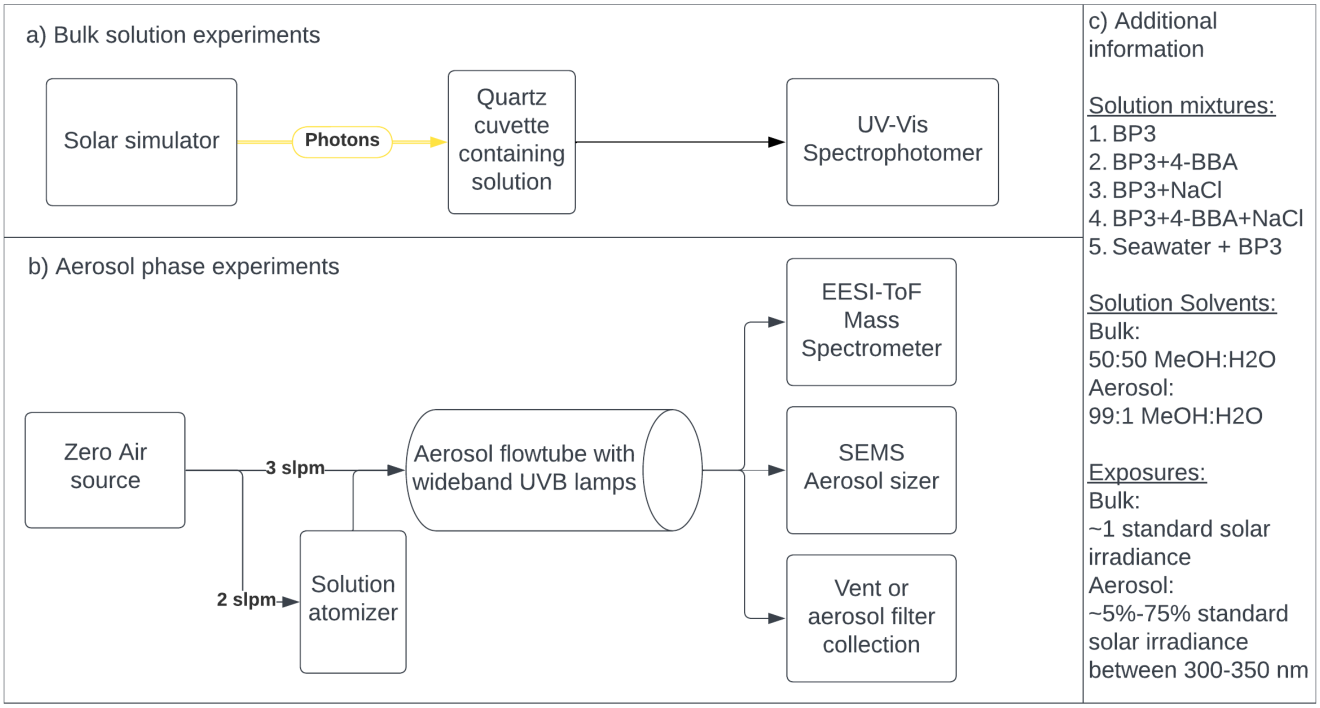

Figure S2. Experimental setup for a) bulk solution and b) aerosol phase photodegradation experiments. Brief experimental details may be found in panel c) and are detailed more in the text.

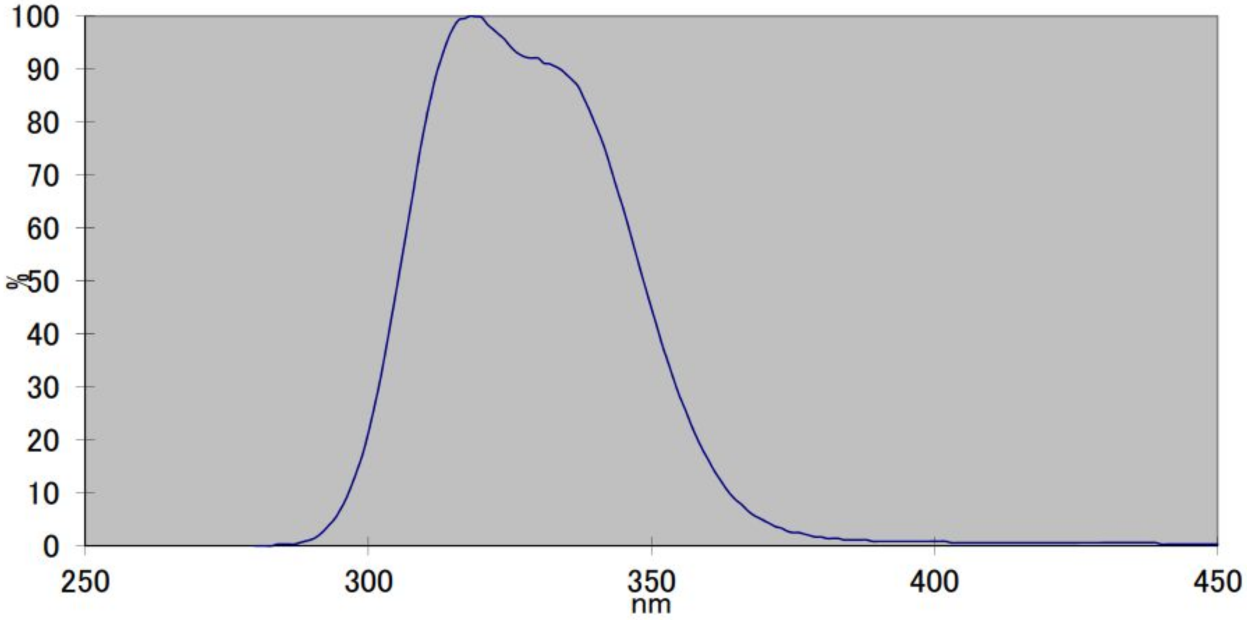

Figure S3. Lamp output spectra of the wideband UVB lamp (provided from manufacturer LCD Lighting.)

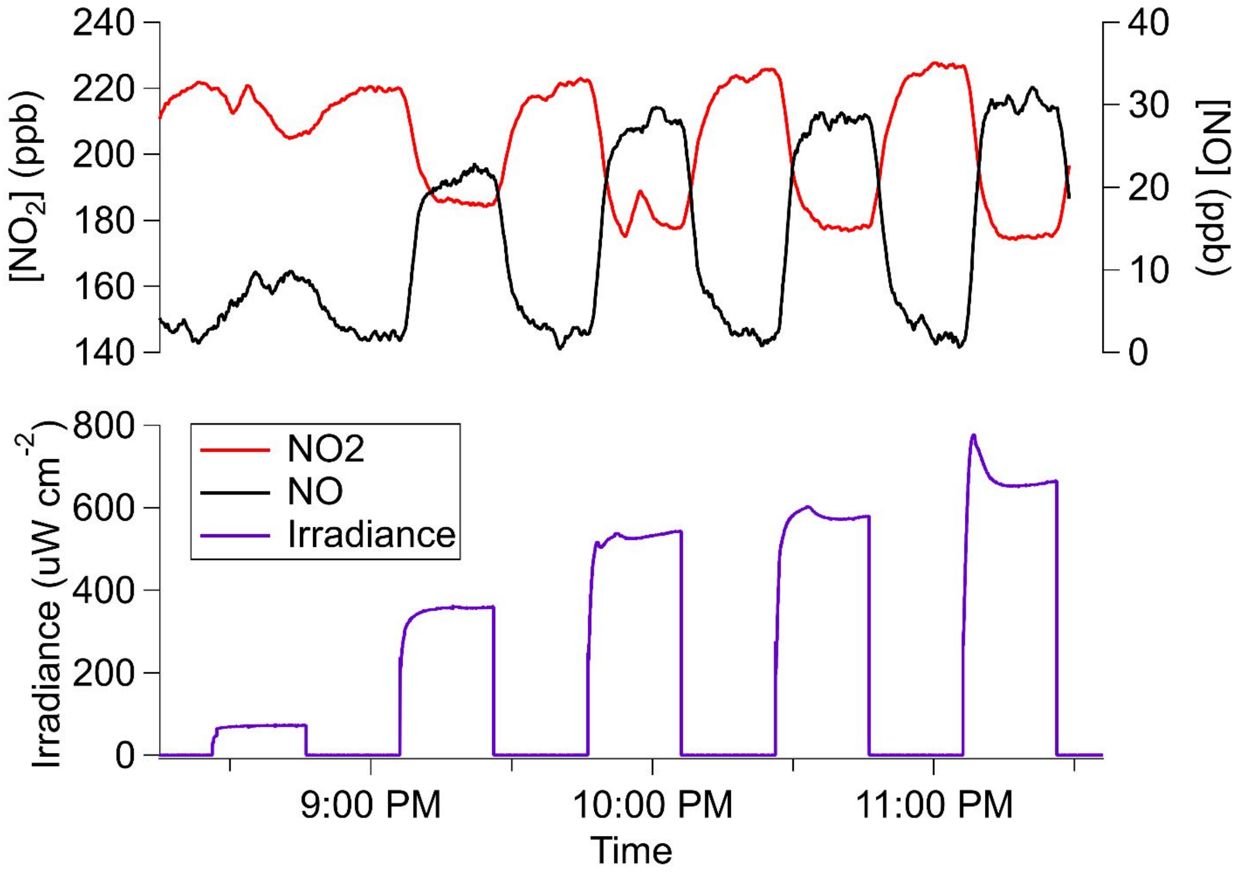

Figure S4. A time series from the calibration of the oxidative flow reactor. a) Concentrations of NO_2 (left) and NO (right) and b) measured irradiance via a photodiode.

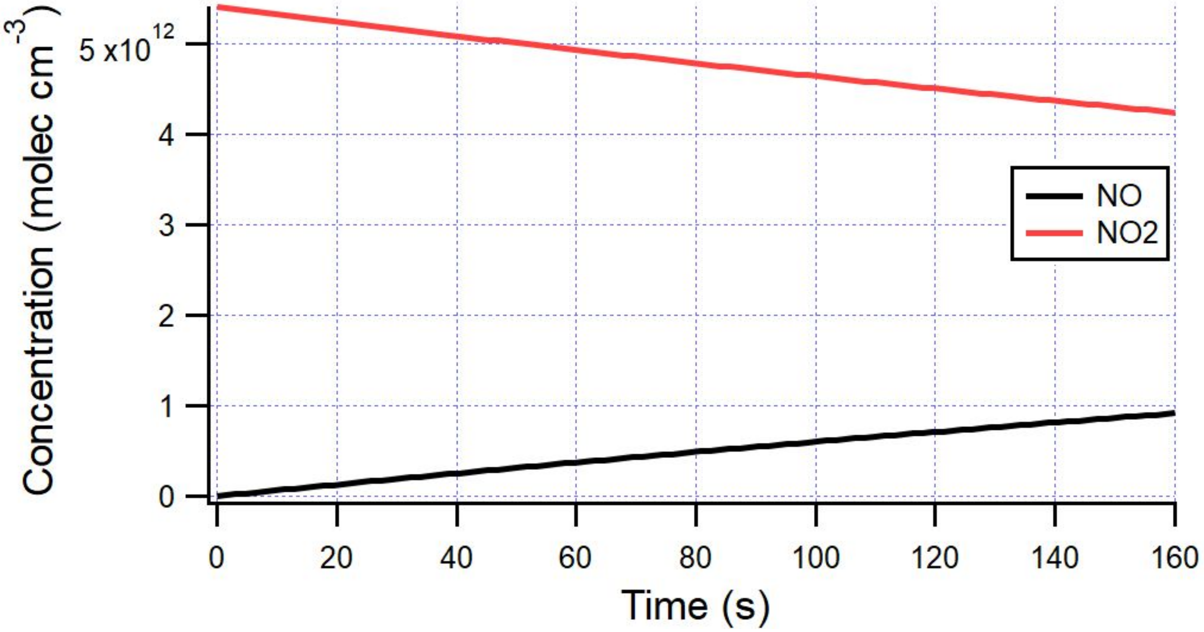

Figure S5. Representative output from a KinSim model used to calibrate observed losses of NO_2 to varying levels of photon fluxes.

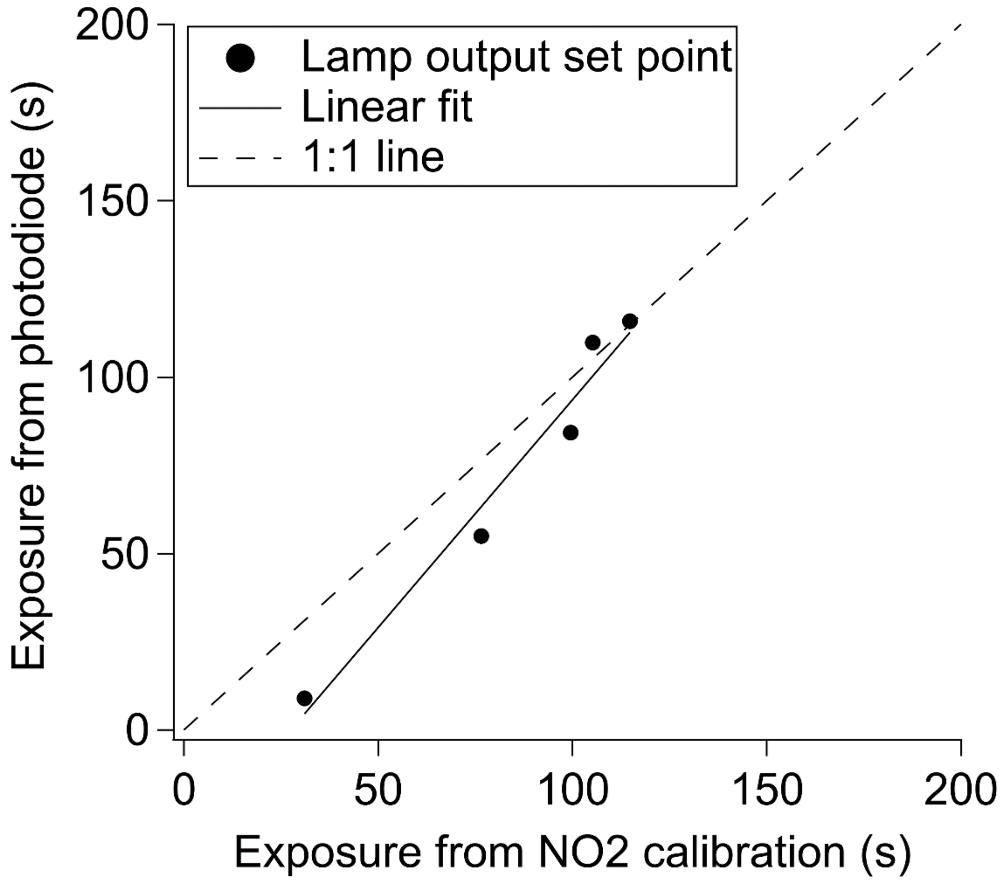

Figure S6. Equivalent solar exposure from calculated NO_2 photolysis rates compared to measurements from a photodiode. The dashed line is a 1:1 fit.

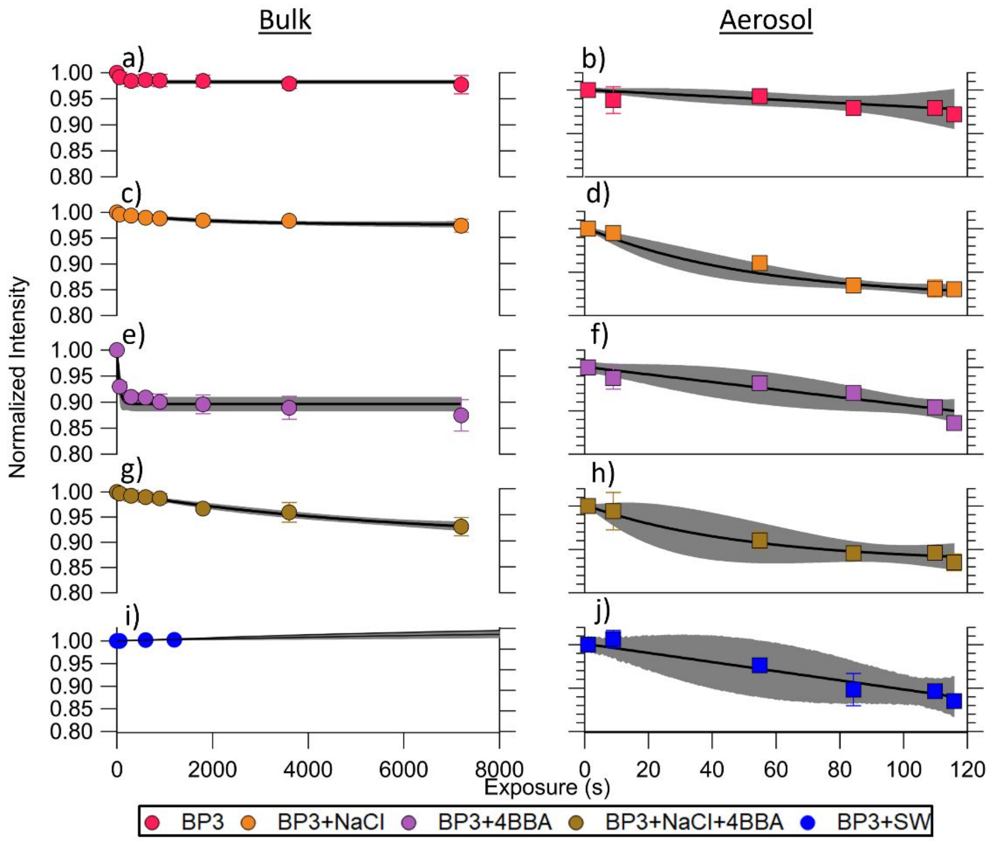

Figure S7. The normalized decay of BP3 over equivalent solar exposure times in solution (a,c,e,g,i) compared to aerosols (b,d,f,h,j). Error bars show one standard deviation from the mean normalized decay for n=3 trials at each lamp intensity, except for the solution trial in seawater, a one-week exposure. Lines of best fit for exponential decay are plotted in black with 95% confidence bands plotted as grey shading.

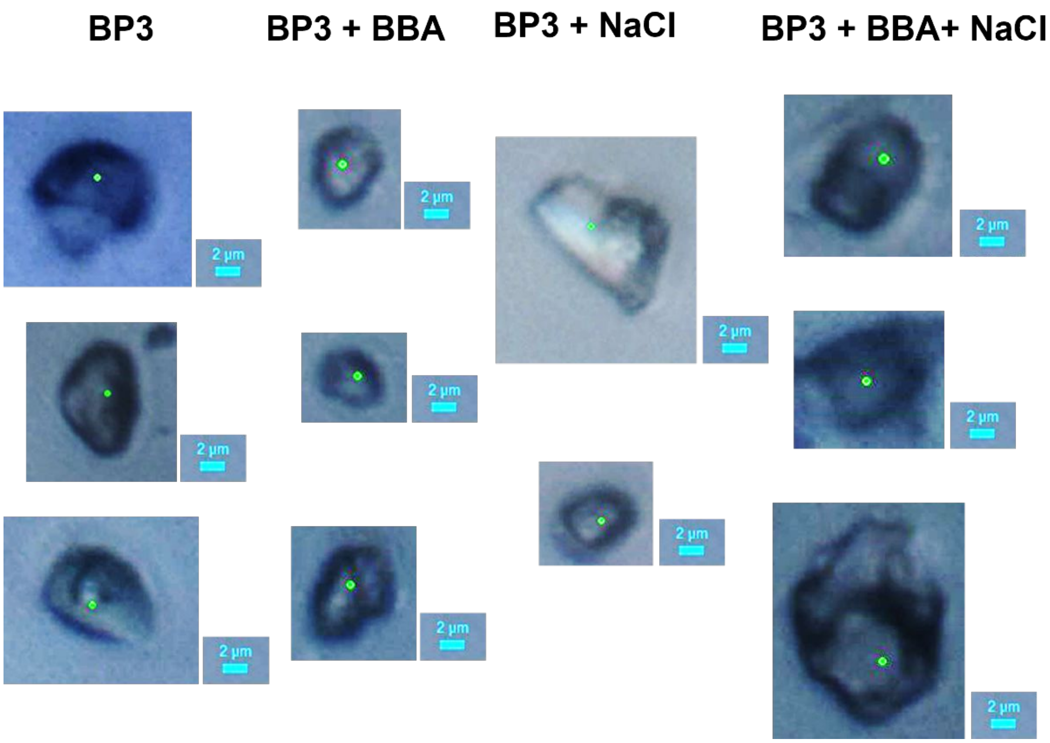

Figure S8. Confocal microscopy photos of each experimental aerosol type deposited onto quartz substrates.

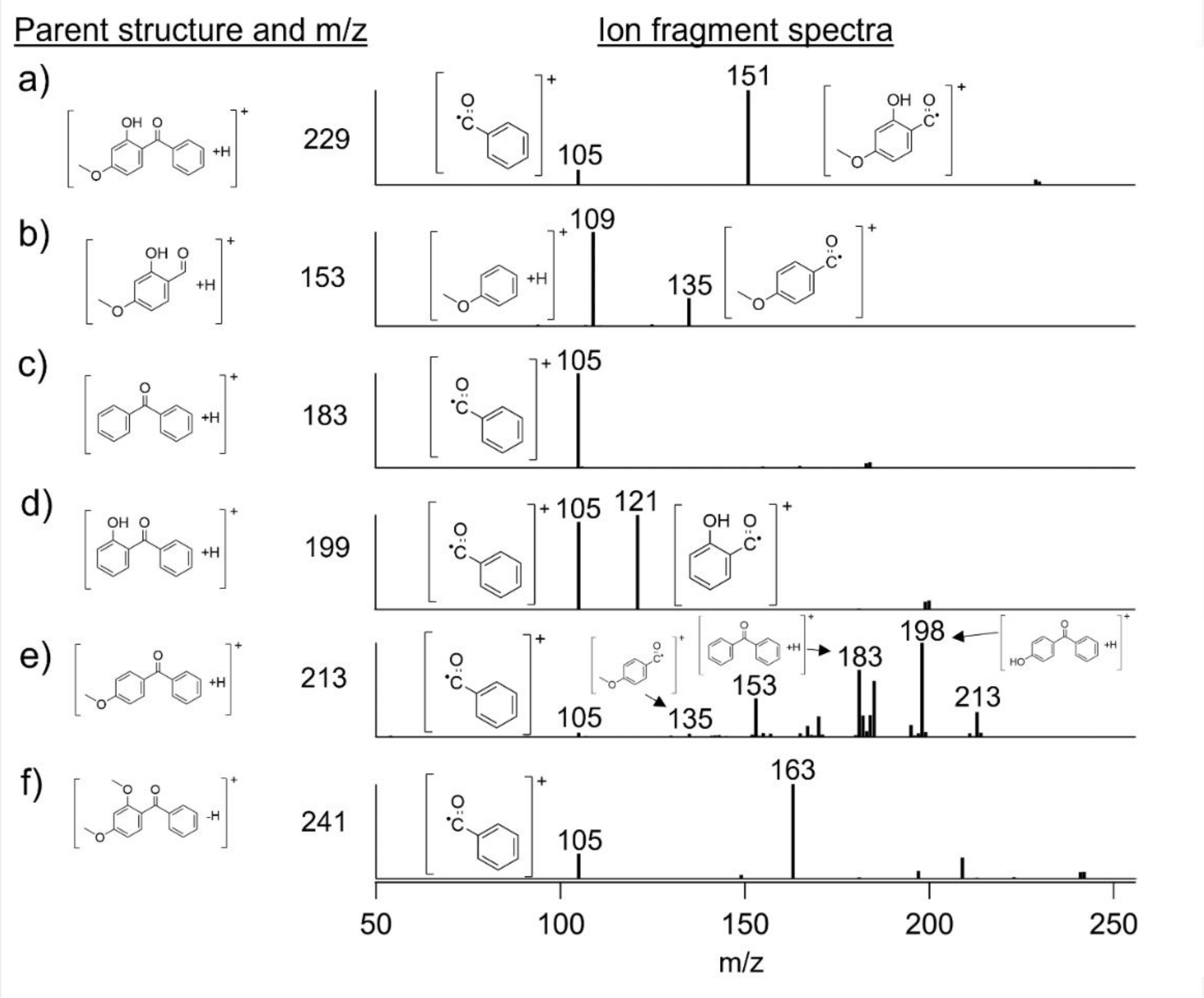

Figure S9. Secondary mass spectra of compounds identified as a) BP3, b) 2-hydroxy-4-methoxy benzaldehyde, c) benzophenone, d) 2-hydroxy benzophenone, e) 4-methoxy benzophenone, and f) 2-4 dimethoxy benzophenone. The numbers refer to the ion's mass-to-charge (m/z) ratio.

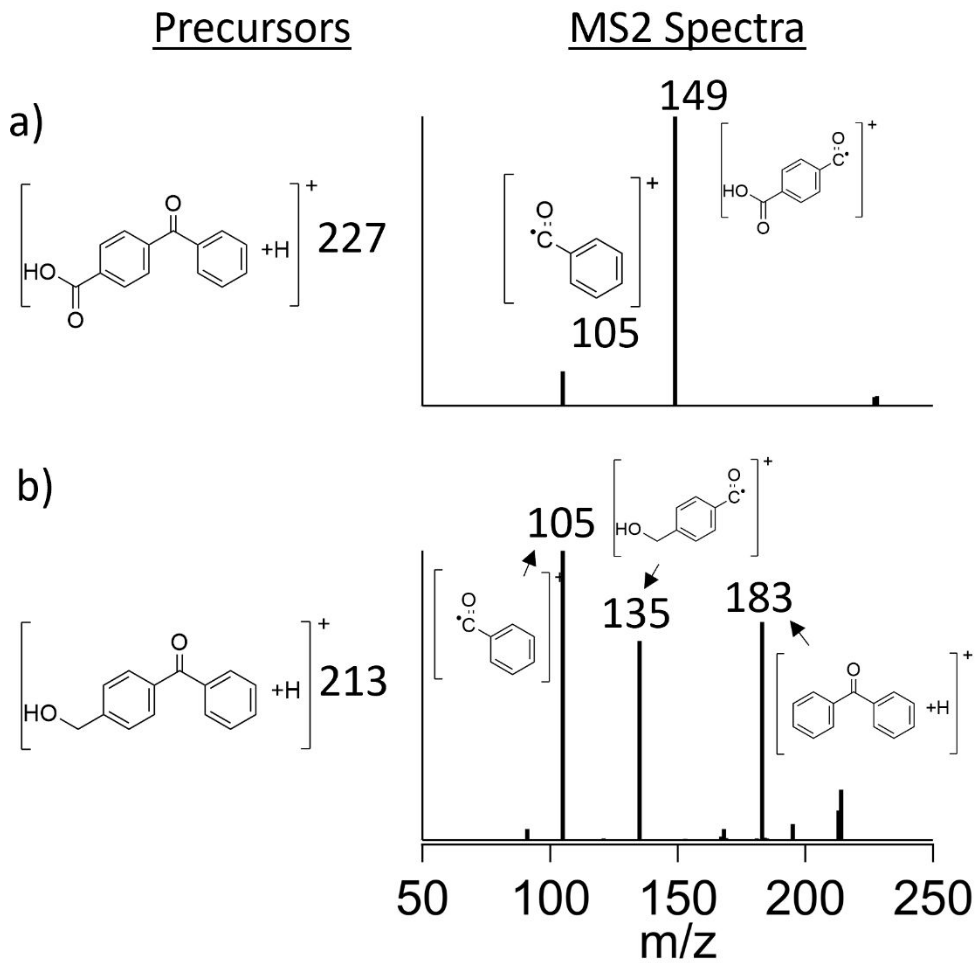

Figure S10. MS2 spectra of a) 4-BBA and b) its major photoproduct, 4benzoylbenzoic methylalcohol.

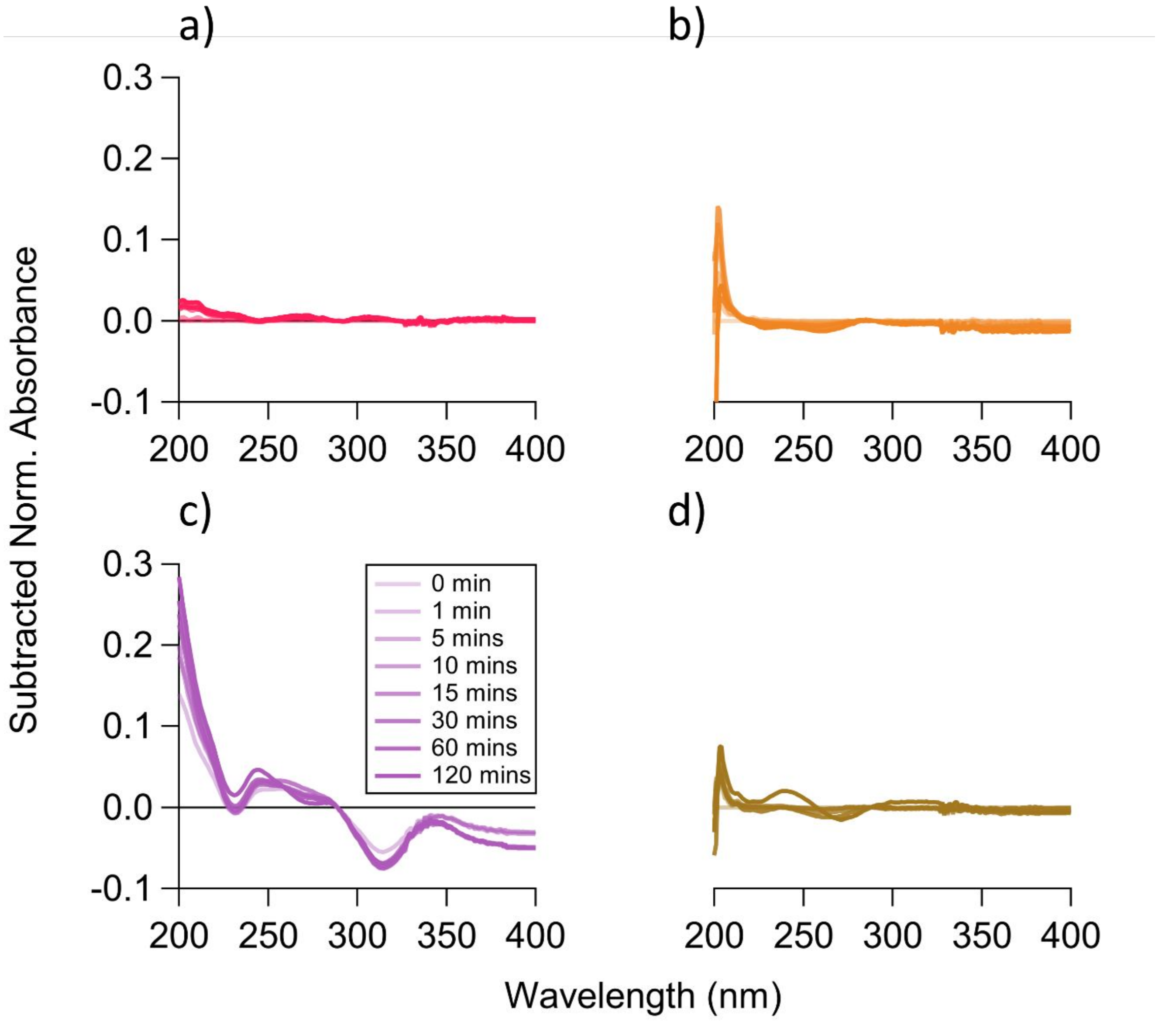

Figure S11. Subtracted UV-Vis spectra of bulk solution experiments normalized to the absorbance at 290 nm for solutions of a) pure BP3 b) BP3+NaCl c) BP3+4-BBA d) BP3+ 4-BBA+NaCl

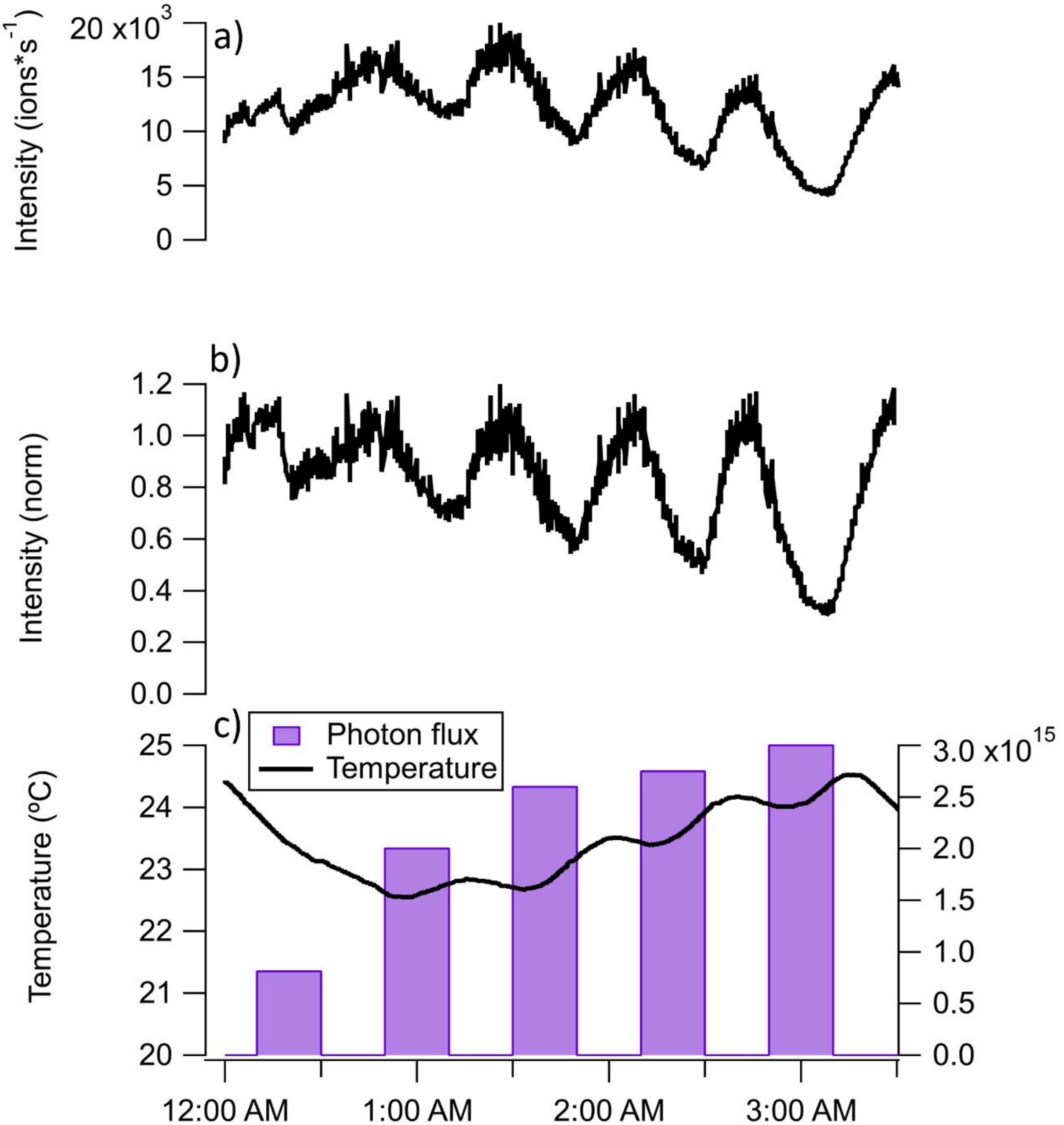

Figure S12. A representative experimental time series for one trial of BP3 decay in pure-component BP3 aerosol. a) Uncorrected measured BP3 intensity (normalized to the TIC and background subtracted). b) Corrected measured BP3 intensity. c) Change in flow tube temperature with varying photon flux.

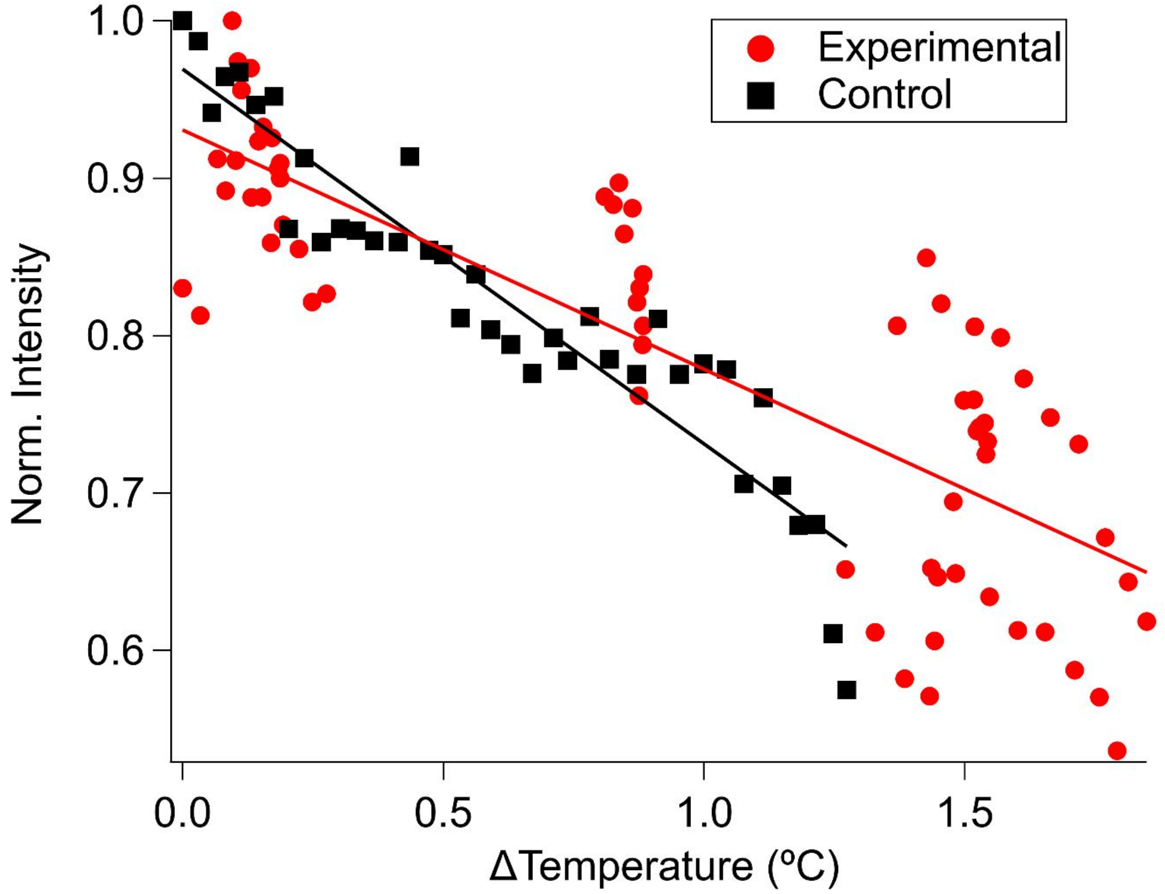

Figure S13. Comparison between temperature-driven changes in signal intensity during experimental trials and a control experiment (where no lamps were used) upon heating.

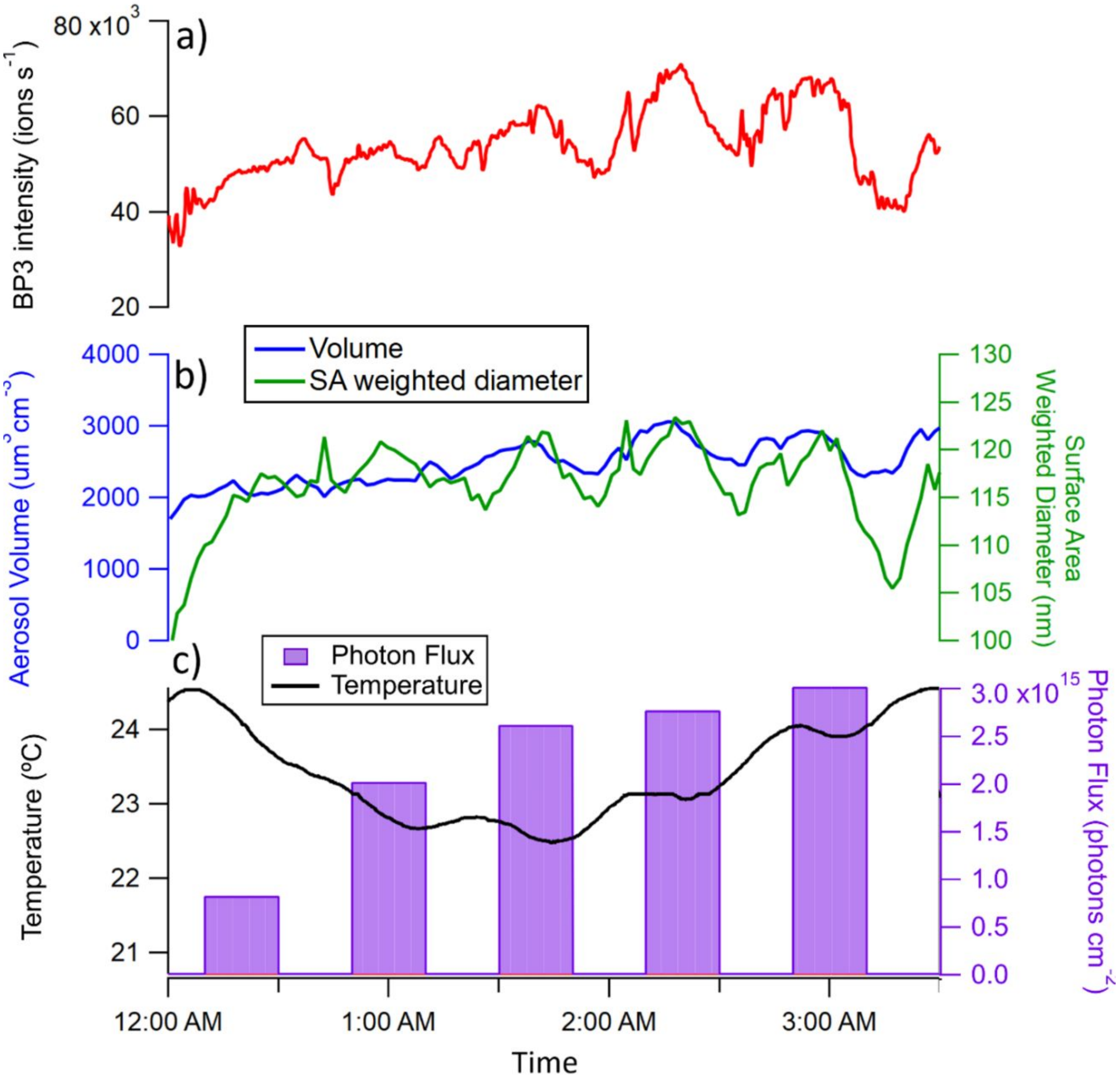

Figure S14. Example experimental trial for BP3 decay in aerosol. a) Uncorrected BP3 mass spectral signal intensity. b) Calculated aerosol volume concentration (left) and surface area weighted diameter (right.) c) Temperature (left) and photon flux (right) in the PAM-OFR.

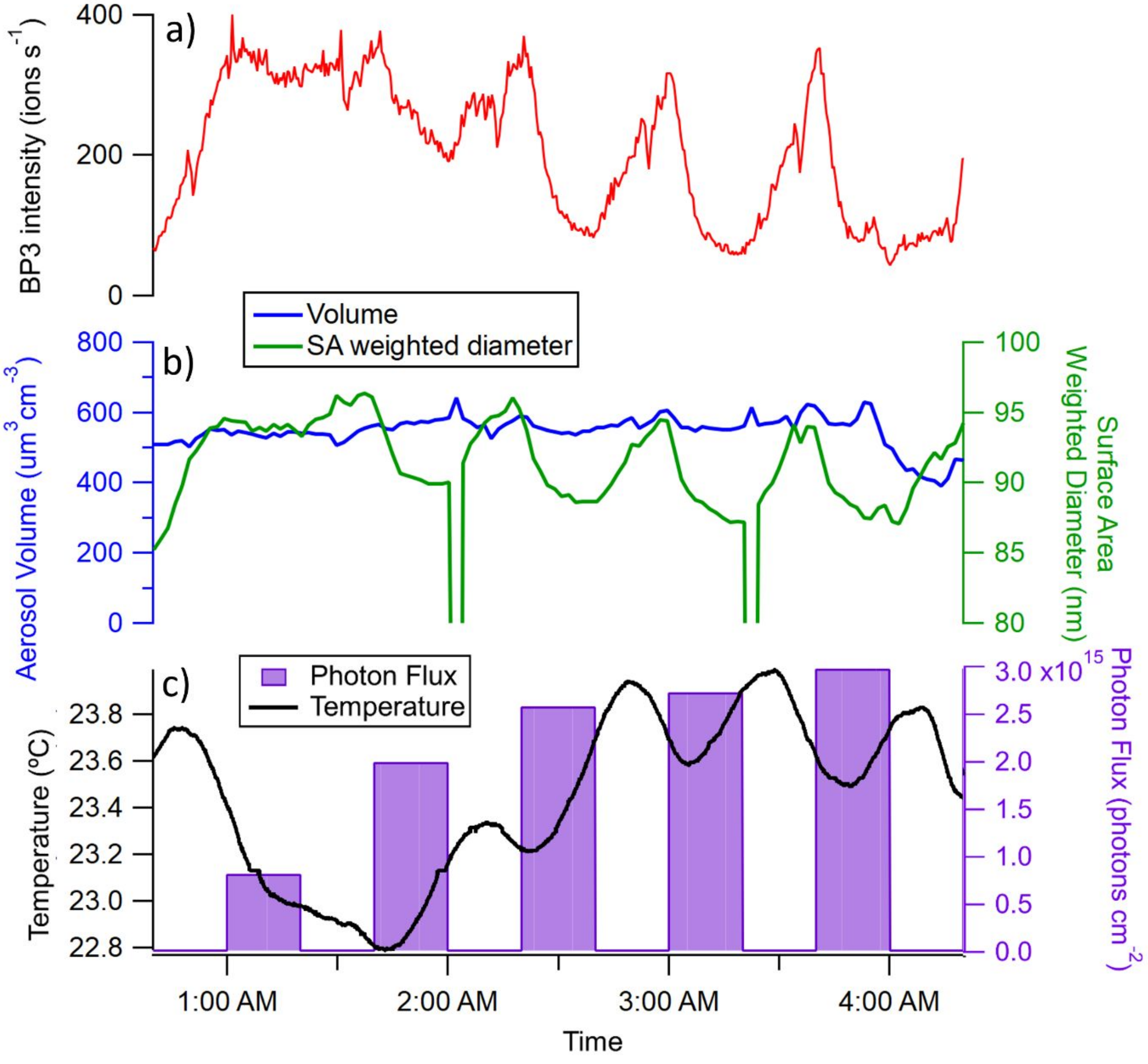

Figure S15. Example experimental trial for BP3 decay in BP3+NaCl aerosol. a) Uncorrected BP3 mass spectral signal intensity. b) Calculated aerosol volume concentration (left) and surface area weighted diameter (right.) c) Temperature (left) and photon flux (right) in the PAM-OFR.

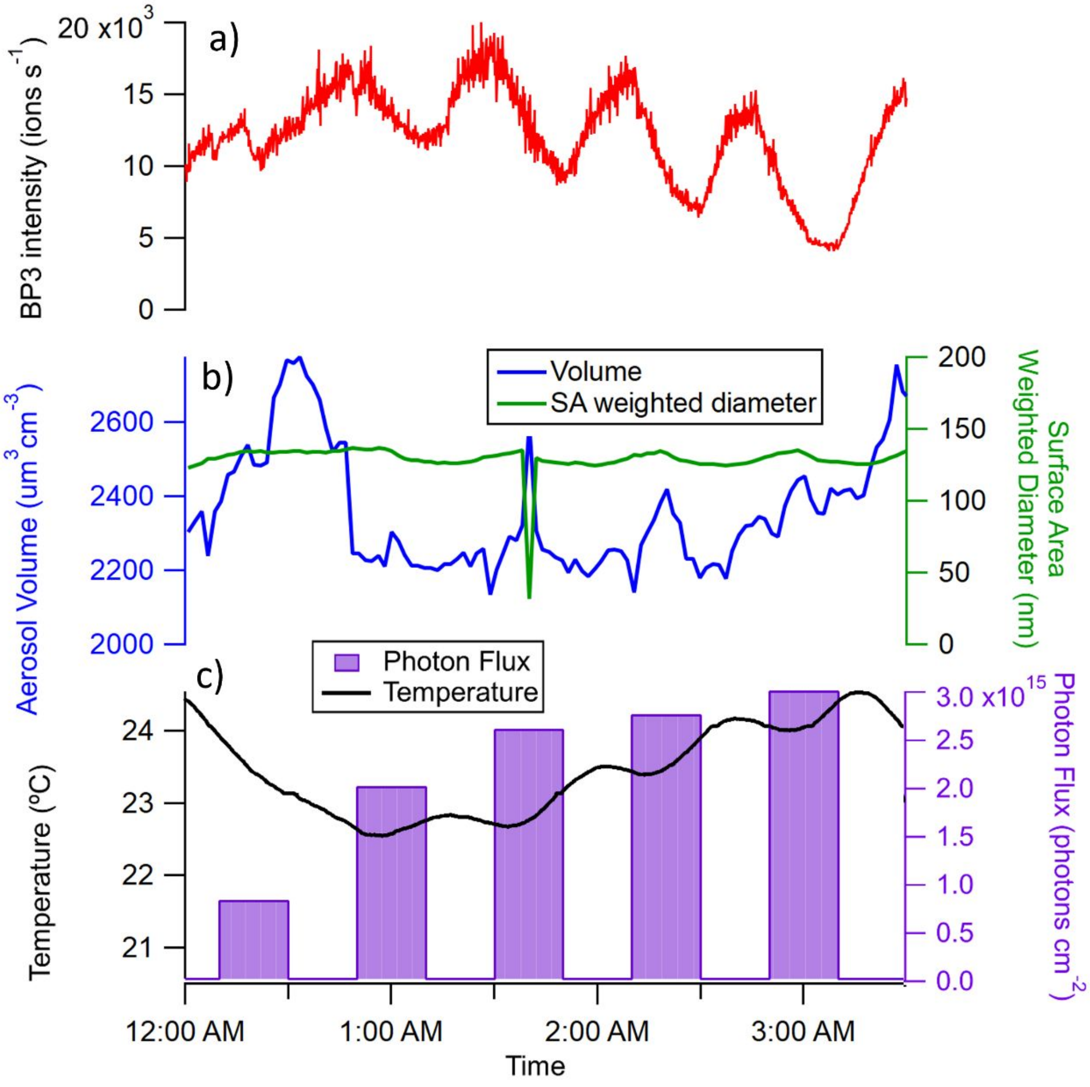

Figure S16. Example experimental trial for BP3 decay in BP3+4-BBA aerosol. a) Uncorrected BP3 mass spectral signal intensity. b) Calculated aerosol volume concentration (left) and surface area weighted diameter (right.) c) Temperature (left) and photon flux (right) in the PAM-OFR.

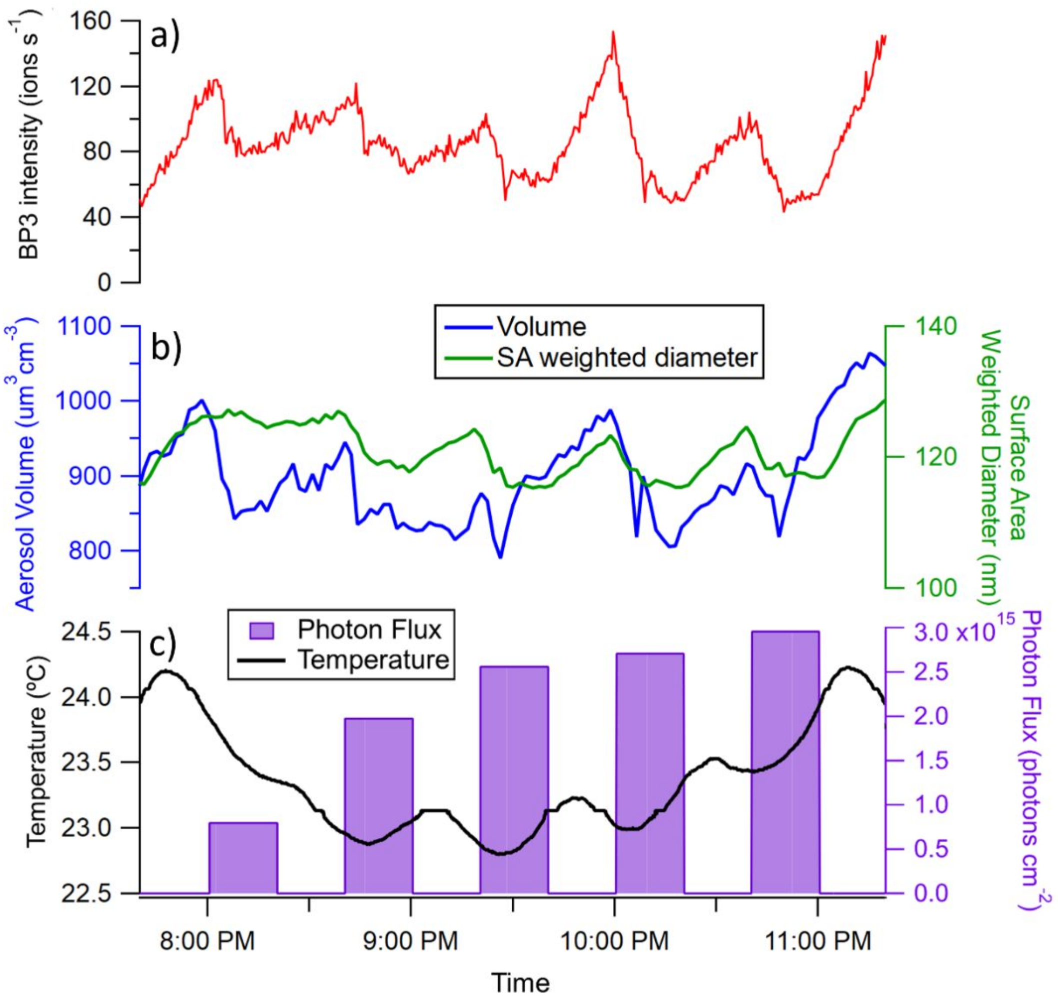

Figure S17. Example experimental trial for BP3 decay in BP3+NaCl+4-BBA aerosol. a) Uncorrected BP3 mass spectral signal intensity. b) Calculated aerosol volume concentration (left) and surface area weighted diameter (right.) c) Temperature (left) and photon flux (right) in the PAM-OFR.

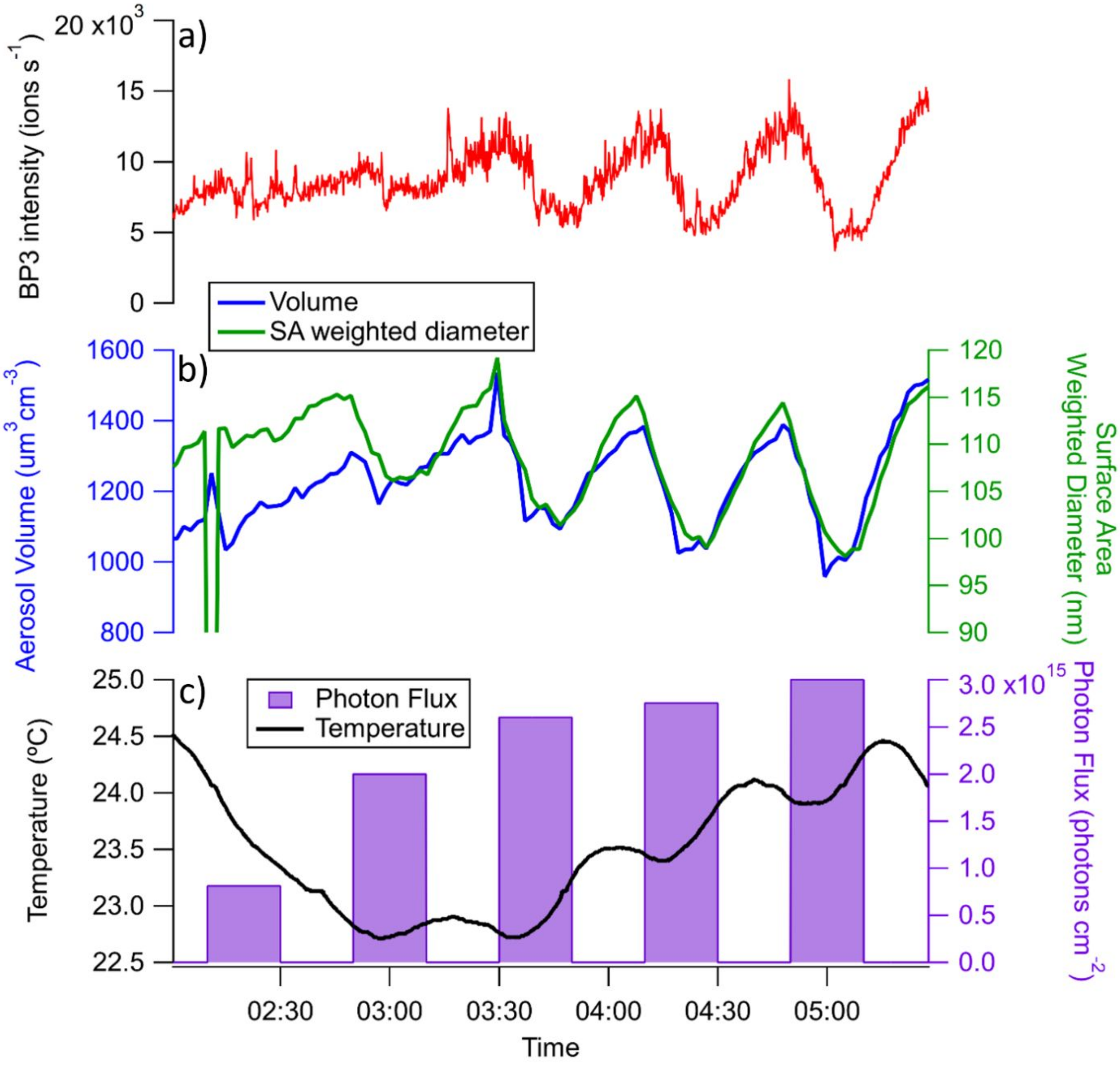

Figure S18. Example experimental trial for BP3 decay in BP3+SW aerosol. a) Uncorrected BP3 mass spectral signal intensity. b) Calculated aerosol volume concentration (left) and surface area weighted diameter (right.) c) Temperature (left) and photon flux (right) in the PAM-OFR.

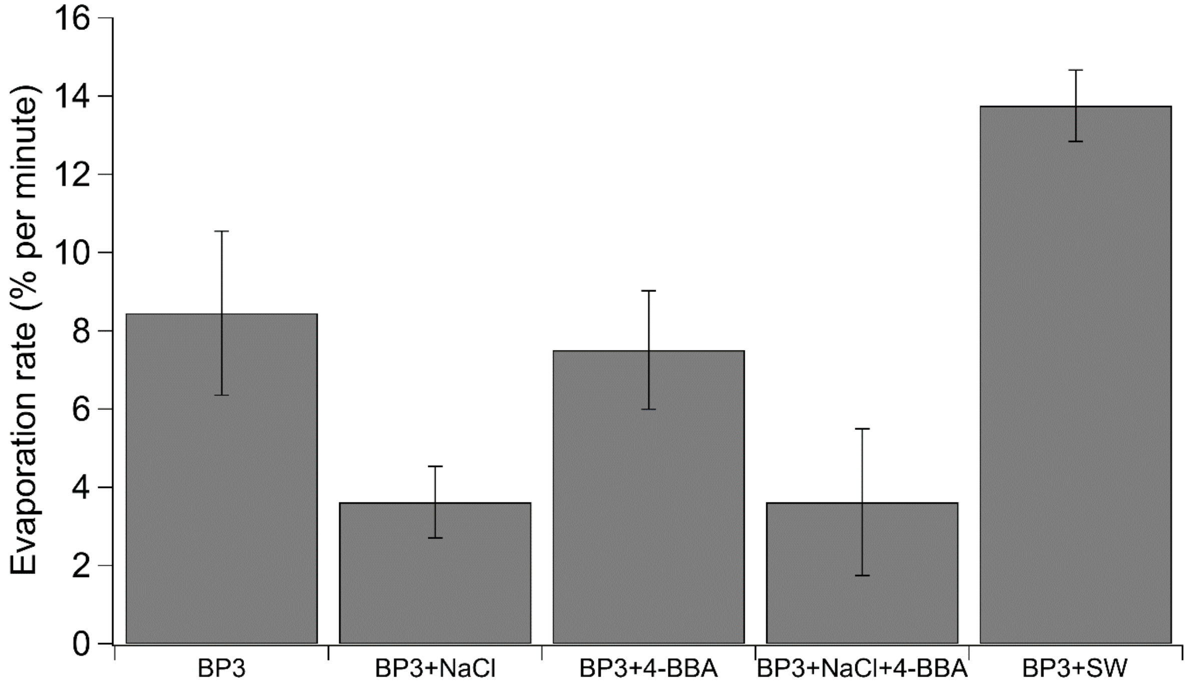

Figure S19. Aerosol evaporation rates for the different types of aerosols. Error represents the standard error in the linear regression mean between the average mass loss as a function of equivalent solar exposure.

Table S1: Experimental conditions for aerosol experiments.

Experiment Number	Solution Type	Solution pH	Relative Humidity	Temperature	Surface Area Weighted Diameter (before exposure)
1-3	BP3	~6.1	~50%-55%	22.5-24.5 C	117 ± 2 nm
4-6	BP3+NaCl	~6.1	~50%-55%	22.5-24 C	95 ± 2 nm
7-9	BP3+4-BBA	~6.1	~35-40 %	22.5-24 C	140 ± 5 nm
10-12	BP3+NaCl+4-BBA	~6.1	~45-50%	23-24.5 C	125 ± 5 nm
13-14	Spiked Seawater	~6.9	~40-45%	22.5-24.5 C	112 ± 5 nm

Table S2: Effective rate constant $J_{eff,env}$ for each bulk solution and aerosol mixture type.

Mixture type	Bulk solution effective rate constant $J_{eff,env}$ (sun^{-1} s^{-1})	Aerosol effective rate constant $J_{eff,env}$ (sun^{-1} s^{-1})
BP3	$(1.7\pm0.6)*10^{-6}$	$(2.3\pm0.3)*10^{-3}$
BP3+NaCl	$(2.5\pm0.4)*10^{-6}$	$(11.0\pm0.5)*10^{-3}$
BP3+4-BBA	$(4.2\pm0.7)*10^{-6}$	$(6.5\pm0.9)*10^{-3}$
BP3+NaCl+4-BBA	$(3.3\pm0.4)*10^{-6}$	$(8.5\pm0.5)*10^{-3}$
BP3+SW	$(0.19\pm0.03)*10^{-6}$	$(6.6\pm0.5)*10^{-3}$

Table S3: Toxicity of BP3 and its photodegradation products estimated by EPA T.E.S.T. Note – these values are reported as negative log values where higher values are more toxic.

Compound name	Experimental or predicted toxicity log (mol/kg) (EPISTAR, LD_{50} rat)	Atmospheric lifetime in the gas phase to hydroxyl radicals (EPI Suite)
Oxybenzone	1.49 (exp)	1.9 hrs
Benzaldehyde	1.91 (exp)	30 hrs
Benzoic acid	1.86 (exp)	9 days
2-hydroxy-4-methoxy benzaldehyde	2.33 (pred)	0.9 hrs
2-hydroxy-4-methoxy benzoic acid	2.27 (pred)	0.9 hrs
Benzophenone	1.58 (pred)	36 hrs
2-hydroxy-benzophenone	1.51 (pred)	4.0 hrs
4-methoxy-benzophenone	1.63 (exp)	5.8 hrs

Supporting Text

UV-Vis Spectra of prepared solutions

For all trials, the absorbance of solutions was dominated by oxybenzone with characteristic λ_{max} peaks at ~289 nm and a shoulder at ~323 nm.[3,53] As such, we expect oxybenzone to be neutral or protonated as the pH of all experiments was ~6-7 (determined from pH strips) and less than its pKa (7.6). Above this pKa, the anionic form of oxybenzone exhibits greatly reduced UV absorbance.[53]

The photosensitizer 4-BBA absorbs around a λ_{max} of ~257 nm. This is blue-shifted from its previously reported absorbance at 264 nm in pure H_2O.[54] We expect 4-BBA to be deprotonated, as the pH of all experiments (~6-7), like pH levels in surface water, was greater than its pKa (3.4-4.85 in different solvents[54]). While care was made to subtract the influence of 4-BBA through blank subtraction, some enhancement in absorbance near its λ_{max} can be seen in solutions containing 4-BBA (the purple and brown traces in **Fig S1**.) As the same concentration of 4-BBA was present in the blank, this could potentially be a product of BP3-4-BBA interaction in solution before irradiation.

Due to the shifting of λ_{max} between the different samples and throughout some experiments, quantification was conducted by integrating within BP3's λ_{max} region between 270 nm and 310 nm, which showed minimal influence in samples containing 4-BBA.

Upon irradiation, all experiments showed a growth in the 200-210 nm wavelength range (shown in **Fig S11**), suggesting the formation of less conjugated products. The

BP3+4-BBA binary mixture (**Fig S11c**) exhibited an additional decrease in the 300-325 nm region and growth in the 240-270 nm region.

Atomized aerosol properties compared to sea spray aerosol

The MeOH in the sample evaporates immediately upon atomization, producing residual dissolved and internally mixed inorganic and organic aerosols.[59] Atomizing aerosols in the lab differs from producing SSA from bubble bursting processes.[43,60] The zero-air carrier flow had an RH of 50%, as measured by an RH probe adapted to the flow tube, which was the same for all experiments. This RH is below the deliquescence RH of pure NaCl (DRH~75%[61]) but above its efflorescence RH (ERH~45%).[61] Internally mixing organic compounds to inorganic aerosol, as done in this study, can allow for water uptake before the DRH. The combination of organic compounds and ammonium sulfate has been shown to reduce particle DRH[62,63] although this is less well explored with aerosols containing NaCl.

We visually inspected the particles' physical characteristics (i.e., morphology) at the same RH as during the experiments using confocal microscopy (Horiba LabRaman HR Evolution) after collecting the particles onto quartz substrates coated in Rain-X. The aerosol morphologies of much larger, micron-sized particles visible under the microscope were consistent with rounded particles for the pure organic aerosols and partially engulfed or phase-separated structures in the mixtures with NaCl (See SI **Fig S8**). In real SSA, the particle morphologies evolve with RH and differ with particle size, exhibiting both rounded and phase-separated morphologies.[60]

Aerosol phase drift correction

Aerosol signal drift was observed throughout each experimental trial and must be corrected before calculating photo-induced degradation kinetics. One representative experiment is shown in **Fig S12. Figure S12a** shows the signal of oxybenzone (detected as its deprotonated form at 227 m/z) throughout five increasing photon fluxes, which are plotted in **Fig S12c.**

Note that the drift in signal maxima while under no irradiation negatively correlates with temperature. This is attributed to evaporation caused by the change in temperature inside the PAM-OFR due to each incremental step up in lamp intensity (**Fig. S12c**). Such temperature deviations inside the PAM-OFR have been shown before to influence aerosol mass.[68]

To account for this drift, we normalized each “dark” period to the level of the first dark period, followed by a linear interpolation between the signals in the dark upon each change in lamp intensity (see **Fig. S12b** for a drift-corrected time series). By performing this drift correction, changes in the normalized signal intensities after correction can be attributed to photo-initiated reactions.

We conducted a controlled study of BP3 evaporative losses in the aerosol phase by heating the OFR wrapped in heat tape in the dark in the presence of BP3 aerosol. This yielded a similar evaporative loss decay rate (24±1% loss per 1°C increase) as observed during the experiments during periods of no light exposure (18±2% loss per 1°C increase), indicating the signal drift was due to loss in aerosol mass from the lamps' heating. See **Fig. S13** for a direct comparison.

Aerosol evaporation

In addition to the changes in aerosol mass due to temperature fluctuations, we observed decreases in mass concurrently with decay in the BP3 signal. This suggests that BP3 transformation products, which may be less volatile than BP3, evaporate from the aerosol upon formation. This phenomenon is well studied from the fragmentation of compounds due to photooxidation.(Lambe et al., 2012; Wong et al., 2015) **Figs. S14-S18** shows representative trials for each aerosol type. The simultaneous decrease in aerosol size and volume (shown in **Figs S14-18b**) corresponds temporally with photon flux but not flow reactor temperature (shown in **Figs S14-18c**).

Aerosol evaporation rates are plotted directly in **Fig S19.** Experimental mixtures containing NaCl exhibited the lowest evaporative losses (~4% min^{-1}), followed by pure organic aerosol (~8% min^{-1}), with the spiked seawater aerosol showing the largest losses (~14% min^{-1}). This suggests that NaCl in aerosol can lead to a stabilizing effect that inhibits the evaporation of organic matter in the particles. We hypothesize that additional, potentially more volatile components of seawater DOM led to the high observed evaporation rates in the spiked trials.

References

(1) Lambe, A. T.; Onasch, T. B.; Croasdale, D. R.; Wright, J. P.; Martin, A. T.; Franklin, J. P.; Massoli, P.; Kroll, J. H.; Canagaratna, M. R.; Brune, W. H.; Worsnop, D. R.; Davidovits, P. Transitions from Functionalization to Fragmentation Reactions of Laboratory Secondary Organic Aerosol (SOA) Generated from the OH Oxidation of Alkane Precursors. *Environ Sci Technol* **2012**, *46* (10), 5430–5437. https://doi.org/10.1021/es300274t.

(2) Wong, J. P. S.; Zhou, S.; Abbatt, J. P. D. Changes in Secondary Organic Aerosol Composition and Mass Due to Photolysis: Relative Humidity Dependence. *Journal of Physical Chemistry A* **2015**, *119* (19), 4309–4316. https://doi.org/10.1021/jp506898c.
